# Integrating GIS and remote sensing for land use/land cover mapping and groundwater potential assessment for climate-smart cocoa irrigation in Ghana

**DOI:** 10.1038/s41598-023-43286-5

**Published:** 2023-09-25

**Authors:** Komlavi Akpoti, Moctar Dembélé, Gerald Forkuor, Emmanuel Obuobie, Tafadzwanashe Mabhaudhi, Olufunke Cofie

**Affiliations:** 1 International Water Management Institute (IWMI), Accra, Ghana; 2 Center for Earth Observation and Environmental Research, Accra, Ghana; 3 Water Research Institute, Council for Scientific and Industrial Research, Accra, Ghana; 4 Centre for Transformative Agricultural and Food Systems, School of Agricultural, Earth and Environmental Sciences, University of KwaZulu-Natal, Private Bag X01, Scottsville, Pietermaritzburg, 3209 South Africa; 5 International Water Management Institute (IWMI), Pretoria, South Africa

**Keywords:** Environmental sciences, Environmental impact

## Abstract

Although Ghana is a leading global cocoa producer, its production and yield have experienced declines in recent years due to various factors, including long-term climate change such as increasing temperatures and changing rainfall patterns, as well as drought events. With the increasing exposure of cocoa-producing regions to extreme weather events, the vulnerability of cocoa production is also expected to rise. Supplemental irrigation for cocoa farmers has emerged as a viable adaptation strategy to ensure a consistent water supply and enhance yield. However, understanding the potential for surface and groundwater irrigation in the cocoa-growing belt remains limited. Consequently, this study aims to provide decision-support maps for surface and groundwater irrigation potential to aid planning and investment in climate-smart cocoa irrigation. Utilizing state-of-the-art geospatial and remote sensing tools, data, and methods, alongside in-situ groundwater data, we assess the irrigation potential within Ghana's cocoa-growing areas. Our analysis identified a total area of 22,126 km^2^ for cocoa plantations and 125.2 km^2^ for surface water bodies within the cocoa-growing regions. The multi-criteria analysis (MCA) revealed that approximately 80% of the study area exhibits moderate to very high groundwater availability potential. Comparing the MCA output with existing borehole locations demonstrated a reasonable correlation, with about 80% of existing boreholes located in areas with moderate to very high potential. Boreholes in very high potential areas had the highest mean yield of 90.7 l/min, while those in low groundwater availability potential areas registered the lowest mean yield of 58.2 l/min. Our study offers a comprehensive evaluation of water storage components and their implications for cocoa irrigation in Ghana. While groundwater availability shows a generally positive trend, soil moisture and surface water have been declining, particularly in the last decade. These findings underline the need for climate-smart cocoa irrigation strategies that make use of abundant groundwater resources during deficit periods. A balanced conjunctive use of surface and groundwater resources could thus serve as a sustainable solution for maintaining cocoa production in the face of climate change.

## Introduction

Cocoa is Ghana’s foremost cash crop and contributed about 3% of the country’s Gross Domestic Product (GDP)^1^ and as much as 20–25% of non-oil export earnings between 2017 and 2020^2^. With about 90% of the total cocoa production coming from smallholder farmers having typical field sizes of less than 4 hectares^1^, cocoa production supports the livelihoods of an estimated four million farming households, contributing to their economic empowerment and the Government’s efforts to reduce poverty in the country. Compared to poverty incidence among non-export farmers (e.g., food crops), poverty incidence among cocoa farmers showed a significant decline from 64% in 1991 to 24% in 2006, which signifies the importance of the cocoa sector to poverty alleviation efforts in Ghana.

However, the sustainability of these gains is threatened by several factors. Among these factors are climate variability and change and associated extreme events (e.g., floods and droughts). As cocoa production in Ghana is predominantly rainfed, unpredictable and erratic rainfall patterns or extremely high temperatures can significantly affect yields and the socio-economic benefits that could accrue to the country and cocoa farmers. Previous studies in several parts of the world have confirmed the impact of climate change on cocoa production and yield^3–5^. For example, Gateau-Rey et al.^3^ observed a yield decline of up to 89% in Brazil due to severe droughts caused by the El Niño-Southern Oscillation (ENSO). For the West Africa region, Ofori-Boateng and Insah^6^ found that extreme temperatures adversely affected cocoa yields and concluded that increasing temperature and declining precipitation trends would reduce cocoa yield in the future. In essence, climate change can reduce the water available for cocoa production through declining rainfall and high temperatures. This calls for identifying and promoting appropriate adaptation strategies to reduce the impact of potential yield declines. Wongnaa and Babu^7^ noted that although cocoa farmers in Ghana are aware of the causes of climate change and its impacts on yield, very few adopt adaptation technologies to build resilience to the potential shocks from climate change.

Adopting climate-smart approaches to large-scale cocoa production may significantly reduce the potential impact of climate change on cocoa production. For example, the possibility of installing supplemental irrigation that supplies the required water in drought situations will forestall the adverse effects of climate change on cocoa production. A study showed that adapting cocoa production in Côte d'Ivoire to climate change through the adoption of irrigation will represent a smart investment that will prevent income losses for farmers^8^. In Ghana and Cote d'Ivoire, irrigation systems (e.g., drip irrigation) are piloted in major cocoa-producing areas.

However, scaling such adaptation technologies depends on critical water resource availability and status information. Accurate information on the availability, accessibility, and quality of water resources must be established as a basis for assessing the feasibility and extent (spatial and temporal) of the implementation of cocoa irrigation. More water resources assessment has become critical due to declining freshwater supplies in the context of increasing population growth and related water demand, increasing pollution of limited freshwater, and increasing competition for water from different economic sectors^9,10^. In Ghana, illegal mining has induced pollution of water bodies in the major cocoa-growing areas^11,12^, limiting surface water availability for cocoa irrigation in some areas. Also, studies have shown high levels of metals, which led to the closure of some water treatment plants^11^ in the cocoa growing belt. In addition, several interventions, including the Government’s industrialization agenda (one district, one factory) and the irrigation and hydropower projects, are set to increase competition for water usage in the coming years.

To assess water resource availability and accessibility, Earth Observation (EO) and geospatial technologies can provide key inputs and information on location, quantity, quality, and fluxes. EO satellites have the peculiar advantage of providing data over large areas and obtaining repeated acquisitions easily compared to traditional mapping approaches. Geospatial analysis techniques such as spatial interpolation can be used to estimate water resource availability in an area based on sampled locations and associated data of existing water points/infrastructure (e.g., boreholes). The use of EO data, especially those with moderate to high spatial resolution, has increased in the last decade due to major space agencies' announcement of open data policies. For example, the USGS’ announcement of an open data policy on its flagship Landsat program has led to a substantial increase in the use of Landsat data for several applications^13^. The European Commission’s Copernicus program, designed with an open data policy, has further enhanced EO data availability for adaptation planning and decision-making in data-poor regions. Compared to Landsat, the Copernicus program provides higher spatial, temporal, and spectral resolution (Sentinel-2). It also delivers Synthetic Aperture Radar (Sentinel-1) data, which, unlike optical data (Landsat, Sentinel-2), is less susceptible to clouds. Additionally, the development of open access algorithms (e.g., QGIS, SNAP) and cloud computing resources (e.g., Google Earth Engine) for processing and analyzing EO data have led to an increase in the use of EO data for resource mapping and environmental monitoring. With geospatial data on existing water resource infrastructure, satellite-based EO data are critical sources for assessing the availability, accessibility, and quality of water resources for cocoa irrigation.

Water resource availability and accessibility can also be assessed based on spatial analysis of water resource infrastructure and/or factors influencing water resource availability. Forkuor et al.^14^ and Akpoti et al.^15^, for example, analyzed borehole data and related attributes, together with factors influencing groundwater occurrence, such as recharge to assess groundwater availability and accessibility for dry season irrigation in the northern regions of Ghana. Influencing factors are derivable from existing geographical data (e.g., geology map, borehole locations, yield) or earth observation and spatial data (e.g., elevation, slope). Spatial analysis techniques such as interpolation and overlays allow the creation and combination of different influencing factors to assess availability and accessibility.

Cocoa production will continue to be central in Ghana’s economy and smallholder farmers' livelihood. Climate-smart cocoa production could be strengthened by adopting and scaling irrigation technologies in the growing areas, creating a buffer against climate shocks. This study was conducted within the framework of the Water, Land, and Ecosystems (WLE)-funded IrriCocoa project to assess the availability and accessibility of various water sources for cocoa production. More specifically, the study used EO and spatial data to assess water availability and accessibility in support of cocoa irrigation in Ghana's cocoa belt. Thus, this paper aims to develop decision-support maps of surface and groundwater irrigation potential using geospatial methods and satellite remote sensing data. The results from this study are expected to guide the government, extension services, and donors in Cocoa irrigation planning in Ghana.

## Materials and methods

### Description of the study area

**Figure 1:**
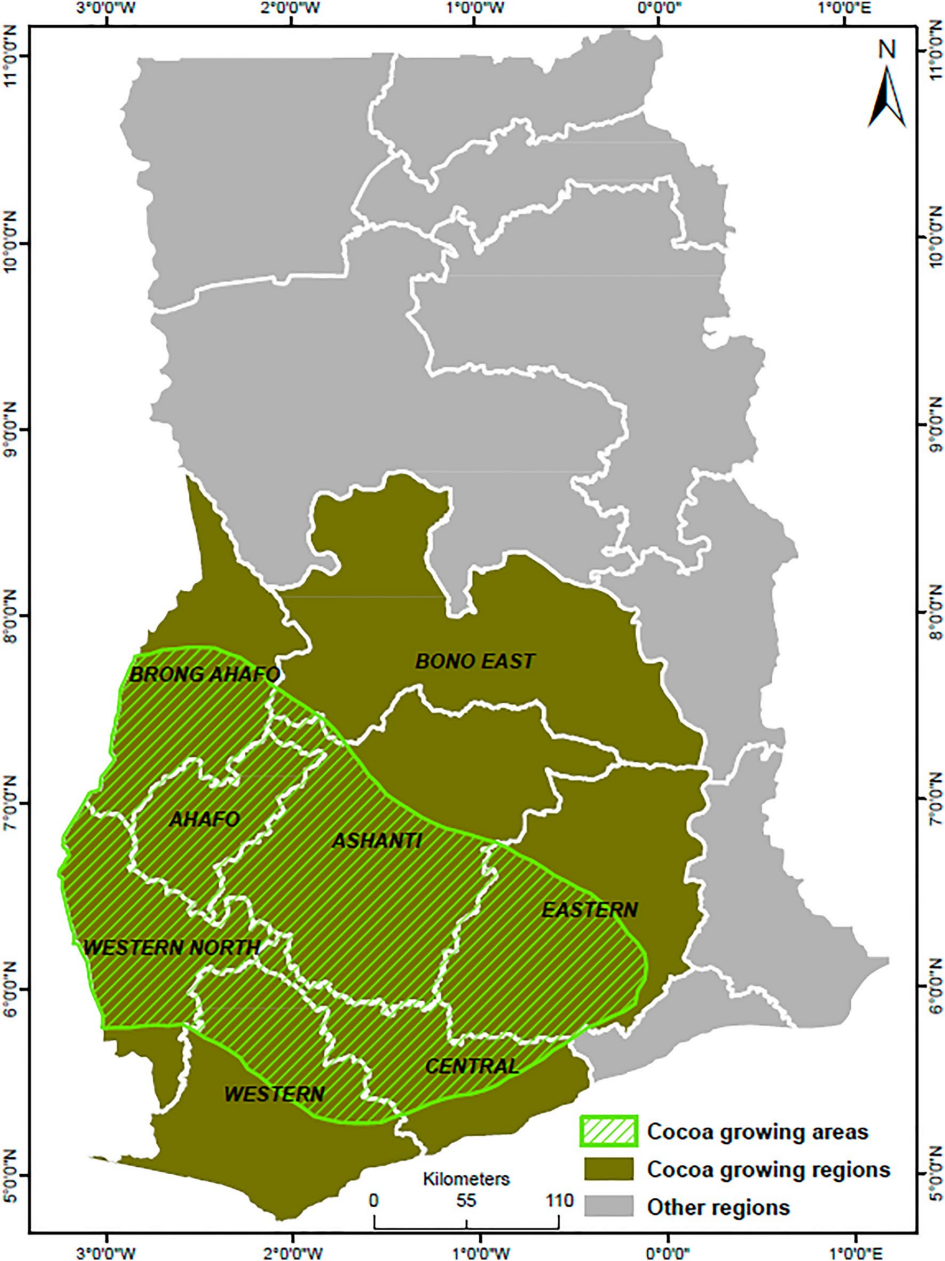
Map of the study area showing the cocoa growing area and administrative regions.

The study was primarily focused on water availability for irrigated cocoa production in the cocoa-growing areas of Ghana (Fig. 1). Ghana’s cocoa belt, located south of the country, lies within eight administrative regions. The growing belt is characterized by a tropical climate, with annual rainfall between 1200 and 2000 mm and annual temperatures between 21 and 32 °C^16^ and high relative humidity. The region has high vegetation cover and tropical forest, making it suitable for cocoa production.

### Remote sensing data and LULC classification

**Figure 2:**
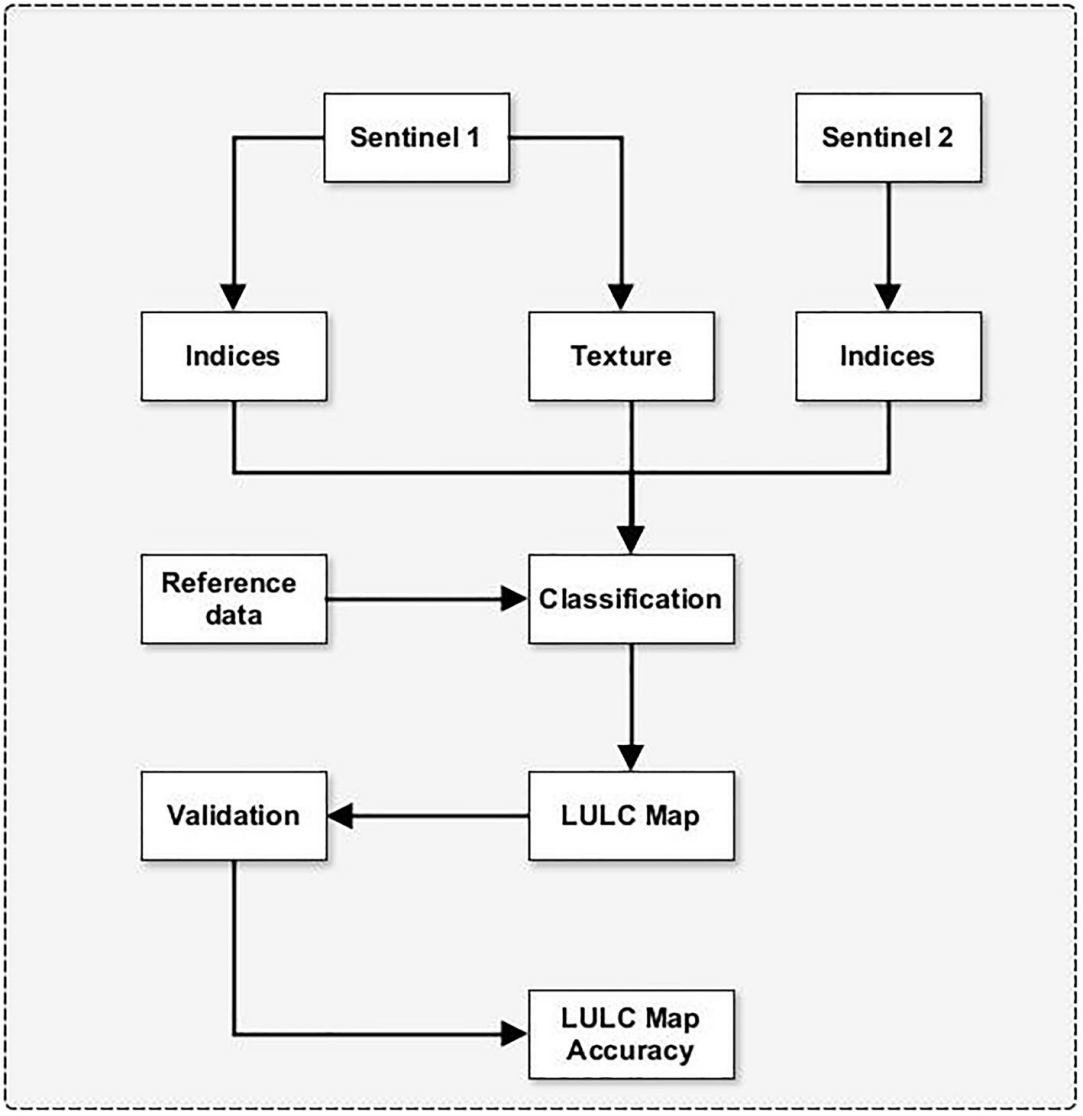
Methodological overview of LULC classification.

Although several global datasets on LULC exist (e.g., notably the 500 m resolution MODIS Land Cover Type Product (MCD12Q1)^17^ the 300 m resolution ESA Climate Change Initiative LULC map^18^ among others), their spatial resolution limits their use for certain local or sub-national analyses. For example, in the framework of the Irricocoa project, two practical challenges are envisaged with the use of these datasets. First, their pixel sizes have a surface area larger than the average sizes of cocoa farms—4 hectares^19^. This means it is difficult to explicitly identify/delineate most cocoa farms. Although there are global datasets at relatively high spatial resolution, e.g., GlobeLand 30^20^, they are old. They have been found to perform poorly in West Africa^21^. Second, the maps do not have a specific class dedicated to cocoa farms or plantations, which can be inferred. This means that even if the spatial resolution is suitable (e.g., 20 m ESA LULC—http://2016africalandcover20m.esrin.esa.int/), they are still not suitable for extracting the location of cocoa plantations. These limitations form the basis of a new LULC mapping effort using moderate to high spatial resolution datasets and the definition of classes dedicated to cocoa and other plantations (See Fig. 2 for the methodological overview).

#### Remote sensing data

Data for the study was acquired from the Sentinel-1 (S-1) and Sentinel-2 (S-2) satellite sensors, accessible via the European Union's Copernicus program. S-1 and S-2 missions, each involving a pair of satellites, provide data that can be used in a complementary way to offer high spatial, temporal, and spectral resolution information for land, water, and atmospheric applications. S-1 is a Synthetic Aperture Radar mission that can always acquire images (day and night) and under all weather conditions. Consequently, S-1 can provide usable images throughout the year to support the analysis of LULC and changes. S-1 operates in four different imaging modes with different spatial resolutions and coverages. Data acquired in the Interferometric Wide Swath (IW) with VV and VH polarizations and spatial resolution of 10 m were used in this study. The S-2 sensors, on the other hand, rely on the sun’s light to acquire images. This means usable images can only be acquired during the day and under clear weather conditions (devoid of cloud cover). Thus, S-2 cannot acquire useful images during excessive cloud cover. This period coincides with southern Ghana's rainy season (April to October). S-2 has 13 spectral bands with multiple spatial resolutions: four visible and near-infrared (NIR) bands at 10 m resolution, six red-edge and shortwave infrared (SWIR) bands at 20 m resolution, and three atmospheric correction bands at 60 m resolution^22^. The visible, NIR, red-edge, and SWIR bands were used. The 20 m resolution bands were resampled to 10 m resolution to ensure optimal integration of the two.

In addition to the spectral bands of S-2 and the backscatter bands (VV and VH polarization) of S-1, spectral indices, and other derivatives were generated as complementary data. Spectral indices and other derivatives have been found to increase classifications' accuracy and improve the separability between land use classes. In the case of S-2, several studies found red-edge dependent indices useful^21^. Textural measures from the Grey Level Co-occurrence Matrix (GLCM)^23^ were computed from S-1 and S-2 as additional layers for the classification. Textural measures have also enhanced class separability and increased classification accuracy^24^. All the eighteen GLCM textural measures were extracted for analysis in Google Earth Engine (GEE)(https://code.earthengine.google.com/), a cloud computing environment hosted by Google Inc. A list of the spectral indices and other derivatives can be found in Table A1.1 in Appendix A1.

#### Ground truth (reference) data

High-resolution Google Earth images of the study area formed the basis for collecting ground truth data. Reference data representing eight LULC classes were collected based on image interpretation and local knowledge. Since images for the nominal year 2020 were analyzed, reference data were collected only from high-resolution images acquired in 2020.

The eight classes considered (and the number of samples collected) are (I) bare land (2708), (ii) cropland—maize, rice, cassava (4763), (3) shrubland (5000), (4) forest (5000), (5) cocoa plantation (5000), (6) other plantations—palm, rubber, teak, orange (5000), (7) settlement—rural, urban (4983) and (8) water—rivers, reservoirs (5000). The locations of cocoa farms were extracted from the website of cocoalife (https://www.cocoalife.org/the-program/approach), which hosts an interactive map of cocoa farm locations in Ghana, Cote d'Ivoire, Indonesia, Dominican Republic, India, and Brazil. The map provides the geographical coordinates and area of cocoa farms in eight out of the sixteen regions of Ghana (see Fig. 1). Using the area of the farms as a guide, the reference data for cocoa were derived by drawing a polygon around the location. Table A1.2 in Appendix A1 lists the eight LULC classes and example pictures from Google Earth that depict them.

#### Land use and land cover mapping

Satellite data processing and analysis were carried out in GEE. These included data search, spatial and temporal subsets, compositing, classification, and accuracy assessment. Using a cloud computing platform eliminates the need to download large-sized EO data for processing on a local disk. It eliminates the need for certain pre-processing steps, e.g., converting digital numbers to reflectance. It provides raw and processed data and automatically merges (stitches) multiple tiles that cover an area of interest. Table 1 details the data types, date ranges, and the number of images (i.e., S-1, S-2) that were acquired for processing and subsequent classification. The median image over the specified periods for S-1 and S-2 were computed from the available image collection, with a maximum cloud cover of 5% set for S-2 images.

**Table 1:** EO data acquired for the classification and its properties.

Sensor/mission	Sentinel-1	Sentinel-2
Data type	Ground Range Detected (GRD)	Multispectral Imager level 2A
Date range	2020-01-01 to 2020-12-31	2019-11-01 to 2020-05-31
Spatial resolution	10 m	10 m
Number of images	452	192
Number of bands/polarizations	10	2
Number of Indices	3	6
Number of textural bands	36 (18 each for VV and VH)	0

Image classification was performed using the Random Forest (RF) Machine Learning Algorithm (MLA)^25^ as implemented in GEE (smileRandomForest). RF belongs to the family of ensemble MLAs that predict a response from a set of predictors by growing many decision trees (forest) and averaging the values predicted by all trees as the final result. The response represents the LULC classes, while the spectral, backscatter bands, and derivatives represent the predictors. Each tree in the forest is independently constructed using a unique bootstrap sample of the training data. In this study, 300 trees were built while all other arguments were set to default. RF is preferred over standard tree-based models because it is less sensitive to noise in the training data and produces more accurate predictive models. Comparative studies involving other MLAs (e.g. SVR, ANN, etc.) also showed RF's superior performance^21,26^. RF is robust against data redundancy and nonlinearity and can handle various predictors with different properties and values^27,28^. An important feature of RF is that it enables a determination of the relative importance of different predictors, which is essential for understanding the data and deciding on data investment.

Before classification, the ground truth data were split into 70% and 30% for training and accuracy assessment, respectively. The classification accuracy was assessed using the 30% to produce a confusion matrix including overall accuracy, producer’s, and user’s accuracy.

### Assessment of the availability of surface water

The assessment of the surface water availability is based on the water bodies detection as classified by the Random Forest algorithm. LULC analysis, described in the previous sections, was conducted to identify, and extract the location of surface water bodies. The surface water, as considered in this paper, includes rivers and reservoirs/dams.

### Assessment of the availability and accessibility of groundwater resources

Availability and accessibility of groundwater resources were conducted due to the increasing limitations of surface water resources. Apart from increasing pollution from activities such as illegal mining and increasing competition from multiple users, the evaporation of surface water bodies reduces the volume of water available for irrigation. Studies in Ghana have found evaporation rates of up to 70%^29^. With temperatures rising and rainfall becoming increasingly erratic, predominantly relying on surface water for irrigation could have dire consequences on the livelihoods of smallholders, food security, economic prosperity, and sustainable development.

Contrarily, groundwater is less susceptible to pollution and evaporation. Although groundwater recharge can be influenced by evaporation resulting in poor infiltration^9,29^, it is generally better buffered against evaporation, droughts, and climate change/variability as compared to surface water^30,31^. Thus, groundwater is a high-potential resource for irrigation. Unfortunately, groundwater is largely underdeveloped, and its use for agricultural purposes lags significantly behind other regions such as China and India^32^. According to FAO AQUASTAT data (https://www.fao.org/aquastat/en/data-analysis/), internally renewable groundwater supplies in Sub-Saharan Africa (SSA) are around 1500 km^3^ per year, a figure that compares favorably with those of China and India, two countries whose agricultural economies have been transformed by groundwater usage^32^. The comparison shows that SSA has about three- and six-times China and India's per capita groundwater availability, respectively^33^. However, high spatio-temporal variability in groundwater availability and accessibility exists, primarily due to high variability in influencing factors such as climate and geology^34^. For local or sub-national assessments, this must be addressed to facilitate decision-making. Explicit delineation of regions with high groundwater availability is critical to identifying areas for cocoa irrigation. Availability in this context refers to the potential quantity or volume of groundwater available in a particular area, but not considering the quality of water.

**Figure 3:**
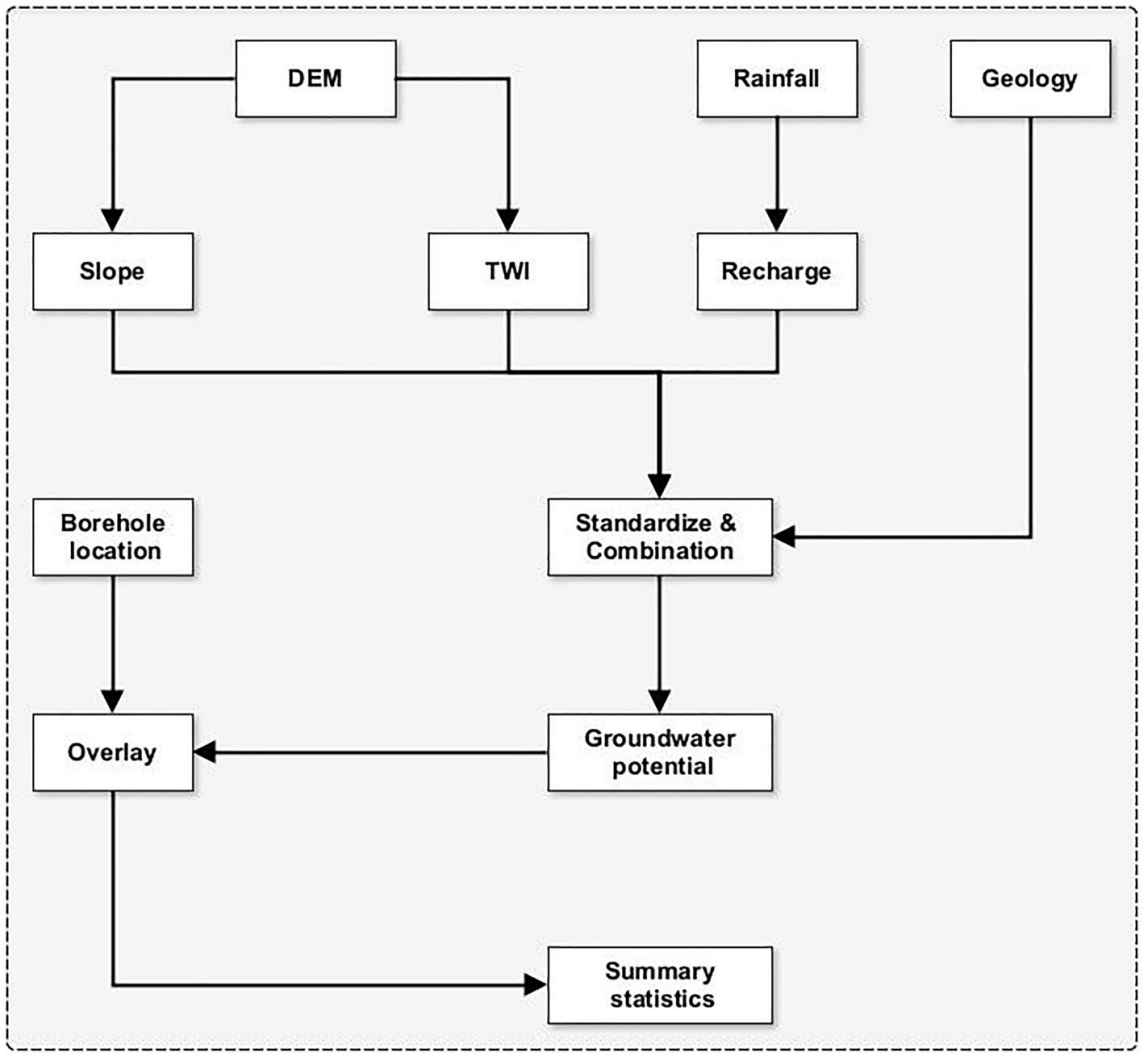
Methodological overview of groundwater assessment.

But it is not all about availability or volumes. The effort required to access the resource is equally important, especially from cost and financial viability perspectives. An optimal solution is identifying areas with high availability but a low cost of accessibility. The next two sections detail the data, pre-processing steps, and methodological approaches employed in conducting the two main analyses (see Fig. 3).

### Data for groundwater resource potential assessment

- Topographic data

Data were obtained from the Shuttle Radar Topographic Mission (SRTM) Digital Elevation Model (DEM) to derive two important terrain-related layers that were subsequently used in the groundwater assessment analysis. These are (i) slope and (ii) topographic wetness index (TWI). The slope is the rate of change in elevation and influences the recharge rate of a region. A low slope has a higher recharge potential, while a high slope drains water faster on the surface and reduces infiltration in the ground (holding soil and geology constant). On the other hand, TWI enables a determination of where water will accumulate in an area with elevation differences. Therefore, areas with high TWI will have a higher recharge potential than areas with low TWI. The formula for calculating TWI is given in Eq. (1):

1 $$TWI=Ln(\frac{a}{tanb})$$

where “a” is the upslope contributing area (flow accumulation) and “b” is the slope in radians.

(b) Climate data

Mean annual precipitation and temperature data between 1950 and 2000 were obtained from the 1 km WorldClim dataset^35^. Temperature was included as a factor in our groundwater potential assessment due to its significant role in the hydrological cycle and influence on groundwater dynamics. Temperature affects evapotranspiration rates, which in turn impact groundwater recharge, and it also influences water demand for agricultural and non-agricultural purposes. Moreover, temperature plays a role in groundwater-surface water interactions, affecting the exchange of water between these systems, which is essential when considering the conjunctive use of surface and groundwater resources for irrigation. Lastly, incorporating temperature as a factor allows us to account for potential climate change impacts on groundwater availability and sustainability in the study area. The precipitation data was used to estimate groundwater recharge and subsequently used to assess groundwater availability. Groundwater recharge is an important determinant of groundwater availability and, therefore, worthy of consideration. Empirical rainfall-recharge equations (Eqs. 2 and 3) were established by Martin & van de Giesen^36^ for the two main geological formations in the Volta basin to derive a rainfall-recharge layer. Although the study area largely falls outside the Volta basin, the geological formations are similar. The geology map of Ghana was first reclassified into two main formations, which formed the basis of applying Eqs. (2) and (3) using map algebra in QGIS.

For weathered (Precambrian) rocks:

2 $$Recharge=0.082*Rainfall-31.141;{R}^{2}=0.747$$

For sandstones (Voltaian) rocks:

3 $$Recharge=0.1316*Rainfall-22.141;{R}^{2}=0.865$$

(c) Hydro-geological data

This consists of borehole locations and a geological map. A national dataset of borehole locations consisting of 4827 records with two groundwater attributes (yield and total depth) was obtained**.** Whereas all 4827 records had yield information, only 1488 had information on total depth. A preliminary assessment of the borehole dataset revealed that multiple records had the same geographical coordinates. The challenge is that multiple records have the same coordinates but different yield values. Therefore, it is difficult to determine how the yields should be assigned to the records with the same coordinates. According to the data providers (i.e., Community Water and Sanitation Agency—CWSA, most boreholes were not geolocated at the drilling time. Therefore, the town’s coordinates where they fall were assigned to them at the time of data digitization. Consequently, multiple boreholes in the same town had the same geographical coordinates.

To resolve this challenge, the average yield of all records with the same coordinates was assigned. This means that only one of the records with duplicate coordinates was maintained, and the average yield of all the records was assigned to it. This can introduce errors, especially for records for which the range of yield values is high. Out of the 4827 records in the national dataset, only 488 unique pairs were realized after the processing. In addition to the national level data, a regional level dataset covering Western and Western North regions (Figure A2.1) had 428 borehole records with additional attributes (e.g., water quality).

A 2010 geology map of Ghana was obtained from the Geological Survey of Ghana. The map consists of the main stratigraphy in the country. The groundwater availability potential of each stratigraphy was assessed based on local knowledge, the number of boreholes, and yield capacity (http://earthwise.bgs.ac.uk/index.php/Hydrogeology_of_Ghana). Table 2 lists the main formations and the ranking given to each concerning groundwater potential. The ranking is from “1” to “10”, where “1” signifies low potential and “10” signifies high potential.

**Table 2:** List of the main stratigraphy in the study area and their rank regarding the potential for groundwater availability*.*

Stratigraphy	Rank
Birimian Supergroup	10
Eburnean Plutonic Suite	7
Tarkwaian Group	3
'Tamnean' Plutonic Suite	4
Dahomeyan Supergroup	5
Buem Structural Unit	6
Voltaian Supergroup, Oti-Pendjari Group	8
Voltaian Supergroup, Obosum Group	1
Voltaian Supergroup, Kwahu-‘Morago’ Group	9
Mesozoic	1
Togo Structural Unit	2

#### Groundwater potential assessment

Three different but related methodological approaches were tested in assessing groundwater availability in the study area. These are: (i) spatial interpolation, where borehole yield was spatially interpolated using geostatistical methods, (ii) regression-based approach, where borehole yield was regressed against groundwater occurrence influencing factors using Machine Learning Approach (MLA), and (iii) multi-criteria analysis (MCA), where groundwater occurrence influencing factors were combined to determine groundwater availability potential zones. However, duplicate records in the borehole data caused the first two methods to produce sub-optimal results. Therefore, this section provides details for only the third approach—MCA, while the first two approaches and results obtained are reported in Appendices A2 and A3.

The MCA approach is premised on the assumption that combining spatial layers of factors or predictors that influence groundwater occurrence or availability enables the identification of high and low groundwater potential areas. MCA outputs indicate a range of suitability or potential (in this case for groundwater availability) from low to high. Forkuor et al.^14^ adopted a similar approach in identifying potential areas for groundwater development for dry season irrigation in northern Ghana. In this case, the output will depict areas of low, moderate, and high groundwater availability potential for the cocoa-growing areas in southern Ghana. The downside of this approach is that it cannot provide a quantitative estimation of groundwater availability. The upside, however, is that it considers multiple factors and guides decision-making on future investments and actions. In the context of the Irricocoa project, an MCA output of groundwater availability can be useful in determining the cocoa farms that are near areas of high groundwater availability potential.

Seven factors were considered in the MCA: (i) groundwater recharge, (ii) geology, (iii) elevation/DEM, (iv) slope, (v) topographic wetness index, (vi) rainfall, and (vii) temperature. Due to the disparate scales (i.e. units) of these factors, they were rescaled between “0” and “1”, with “0” indicating areas of low groundwater availability potential and “1” indicating high potential. This was achieved using fuzzy set theory (Malczewski 1999). Three fuzzy membership types implemented in ESRI’s ArcGIS were used:

- *Small* where small values of the input layer have high membership in the fuzzy set and vice versa. A membership value of 0.5 is assigned to the midpoint, which by default is the midpoint of the range of values. The elevation, slope, and temperature layers were rescaled with this fuzzy membership type;
- *Large* large values of the input layer have high membership and vice versa. A membership of 0.5 is assigned to the midpoint. The recharge, TWI, and rainfall layers were rescaled with this fuzzy membership type;
- *Linear* membership is calculated based on the linear transformation of the input raster. A membership value of 0 and 1 are assigned to the minimum and maximum values of the input layer, respectively. The geology layer was rescaled with this method.

**Figure 4:**
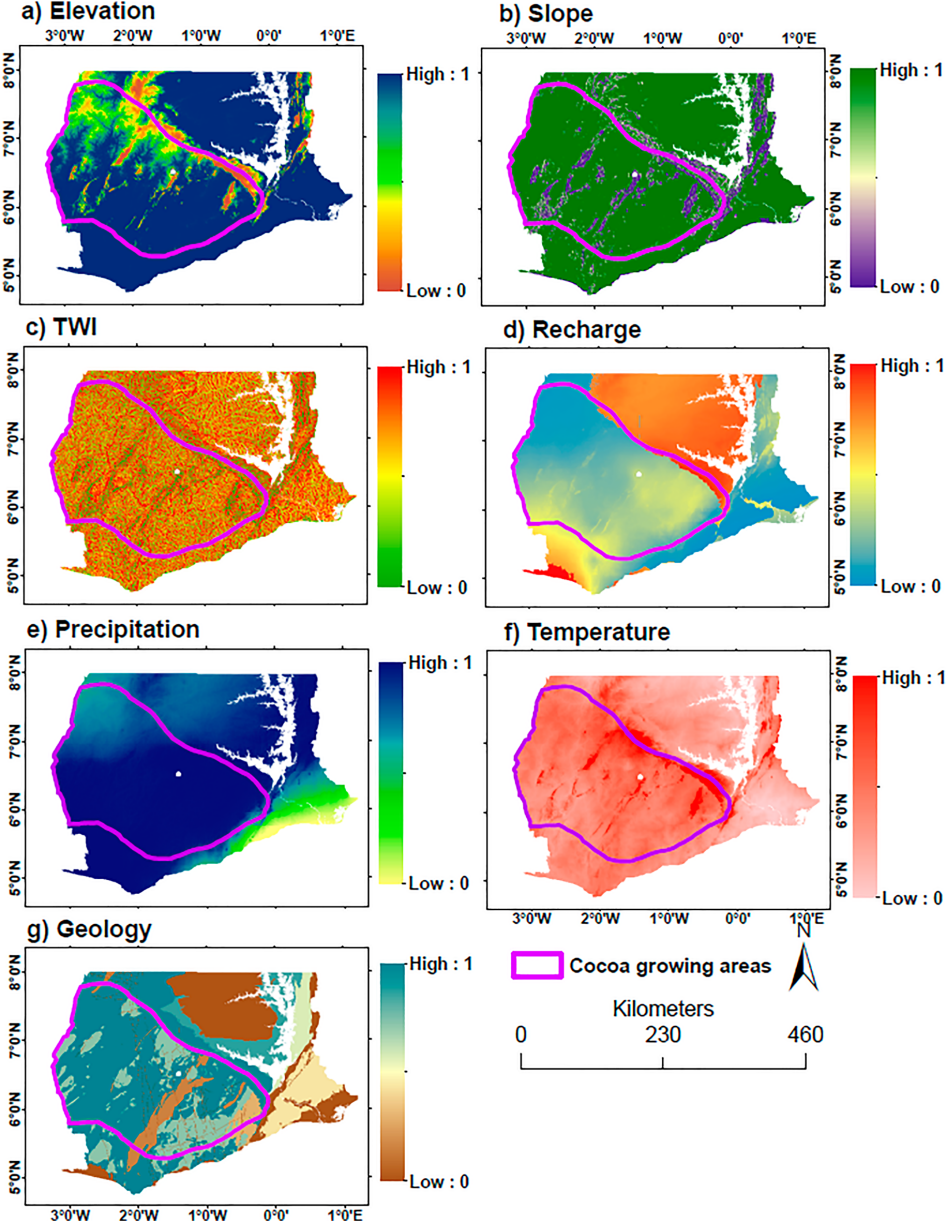
Rescaled maps of groundwater occurrence influencing factors.

The rescaled maps are shown in Fig. 4.

*Combining the factors* The rescaled groundwater occurrence influencing factors were combined using a simple arithmetic mean following Eq. (4).

4 $$P={\sum }_{i=1}^{n}WiXi$$

where “P” is groundwater availability potential, “Xi” represents factor “i”, “Wi” is the weight of factor “i”, and “n” represents the number of factors.

Saaty & Tran (2007) presents a procedure—Analytical Hierarchical Procedure—to objectively determine the weights of influencing factors before combining them. Since the subjective determination of weights would have no scientific basis, all influencing factors were considered equally important and assigned equal weights. Note that the weights sum up to 1 (i.e. 0.143 for each of the seven factors). The resulting map was classified into five classes using natural breaks in the layer’s histogram.

*Test of reliability* To measure the reliability of the resulting groundwater potential map and the potential quantities (yields) obtainable, it was overlaid with the locations of existing boreholes. This overlay enabled a determination of the number of boreholes that exist in each of the modeled potential zones. It is assumed there will be more existing boreholes in high groundwater availability zones than areas modeled as having low potential. Consequently, the overlay was conducted to determine the number of boreholes and the minimum, maximum, mean, and standard deviation of borehole yields in each modeled potential zone. It is further assumed that the average yield of boreholes falling in each potential zone can be considered a quantitative measure of groundwater availability in that zone.

### Assessment of groundwater availability through anomaly-based analysis using GRACE and GLDAS Data

Furthermore, we conducted a temporal and geospatial assessment to monitor changes in groundwater in the cocoa-growing regions of Ghana, by leveraging the Gravity Recovery and Climate Experiment (GRACE) and the Global Land Data Assimilation System (GLDAS) dataset provided by the GRACE Groundwater Subsetting tool (GGST)^37^ under the NASA SERVIR program’s API and Google Colab Notebook. The Groundwater storage anomaly in the database is derived using the mass balance approach (Eq. 5):

5 $$GWa = TWSa{-}\left( {SWa + CANa + SMa} \right)$$

where GWa is the derived groundwater anomaly, TWSa is the GRACE total water storage anomaly (TWSa) derived from GRACE’s monthly mass grids at 3 × 3 degrees, while the surface water anomaly (SWa), canopy storage anomaly (CANa), and soil moisture anomaly (SMa) are derived based on GLDAS Data. These variables were computed based on the monthly outputs from three embedded land surface models (Noah, VIC, and CLSM) as explained in^39^. This allows to compute the uncertainties associated with the data as in Eq. (6):

6 $$GWa= \sqrt{{\left(\sigma TWSa\right)}^{2}-{\left(\sigma SWa\right)}^{2}-{\left(\sigma CANa\right)}^{2}-{\left(\sigma SMa\right)}^{2}}$$

where $\sigma SWa$, $\sigma CANa$, and $\sigma SMa$ are the standard deviations of each component and σTWSa is the total water storage uncertainty provided by NASA^39^.

Each variable in Eq. (6) was assessed, and a time-series plot was created, showcasing the monthly data spanning from January 2003 to December 2020 along with the uncertainty band based on zonal mean over the cocoa growing area. In addition, long-term monthly mean anomaly spatial plots were created to visualize the spatial and seasonal variability of groundwater as well as the mass balance components in the cocoa-growing regions in Ghana. Note that the gaps in the GRACE total water storage anomaly (TWSa) were imputed based on the methods described in^37,38^ and the snow water equivalent is zero for the region.

## Results and discussion

### Land use and land cover mapping

Table 3 shows the classification accuracy matrix derived from the validation of the classified map. The overall accuracy obtained is 96.8%, above the minimum of 75%, which is normally considered acceptable or accurate^40^. Good classification accuracies were obtained for all classes, although some confusion between the vegetative classes is evident.

**Table 3:** Classification accuracy matrix.

	Bare	Shrubland	Cropland	Forest	Cocoa	Plantation	Settlement	Water
Bare	815	9	5	0	0	0	5	0
Shrubland	3	1381	22	0	25	3	0	0
Cropland	0	11	1432	1	24	18	0	0
Forest	0	3	4	1419	41	22	0	0
Cocoa	0	20	28	17	1361	13	0	0
Plantation	1	5	6	4	21	1444	0	0
Settlement	5	0	0	0	0	0	1493	0
Water	0	0	0	0	0	0	0	1475
Total	824	1429	1497	1441	1472	1500	1498	1475
Prod. Acc (%)	98.9	96.6	95.7	98.5	92.5	92.3	99.7	100

In particular, shrublands were misclassified as cropland or cocoa plantations. Crops such as cassava and maize could easily be confused with shrubs if the image temporal series is insufficient to detect temporal changes in their growth patterns. Some young plantations could also be confused with croplands at senescence or full maturity stage. There were also misclassifications between cocoa, on the one hand, and shrubland, cropland, forest, and other plantations, while some forest areas were misclassified as cocoa or other plantations. The accuracies obtained are good and can form the basis of preliminary decision-making. However, ground truthing of the map is recommended to further ascertain its accuracy.

**Figure 5:**
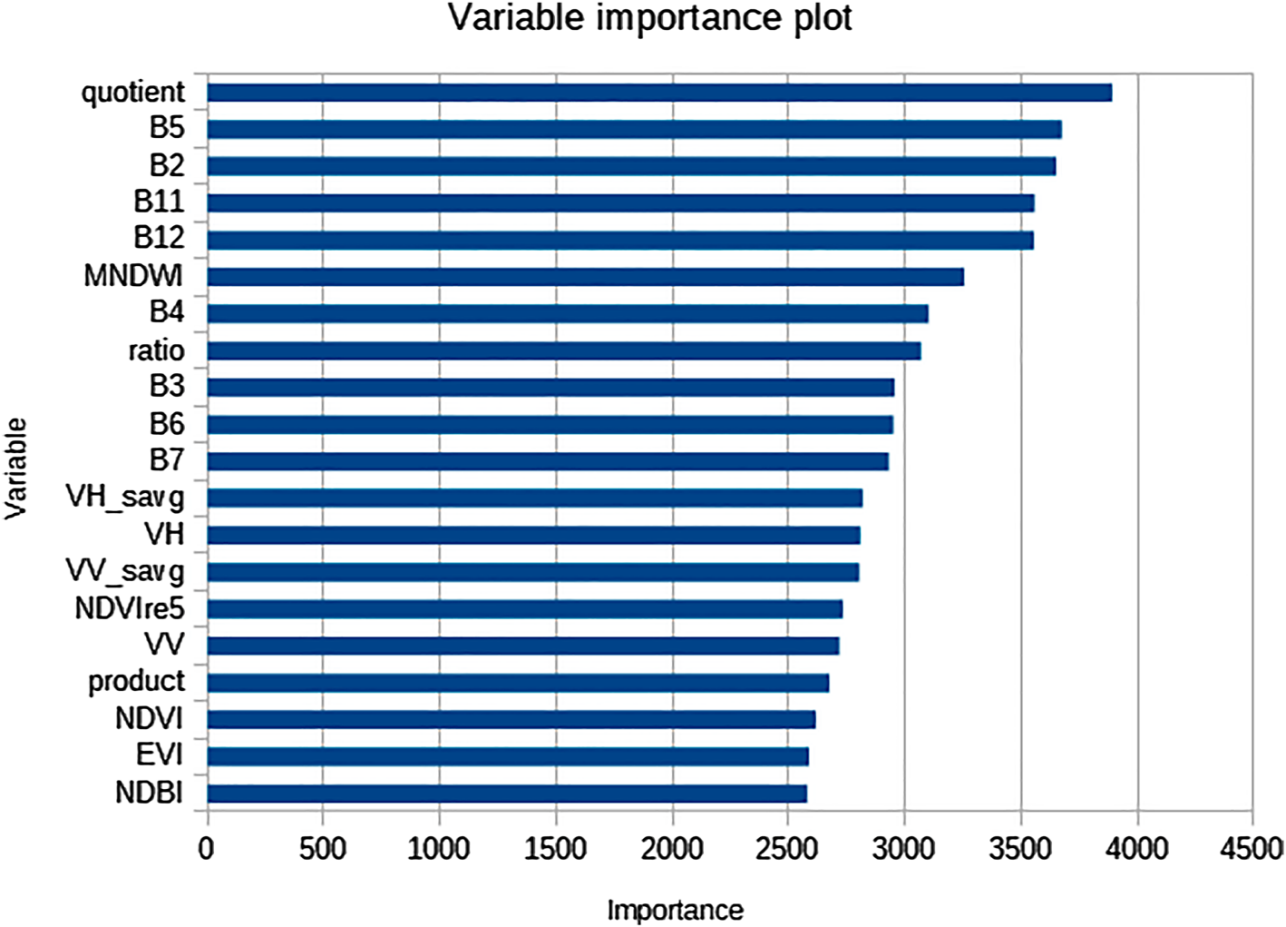
Variable importance plot computed from the classification of LULC classes in cocoa growing areas in Ghana.

In our LULC classification, the importance of sensing variables played a crucial role in accurately identifying and distinguishing between the classes of Bare land, Shrubland, Cropland, Forest, Cocoa, Plantation, Settlement, and Water. Figure 5 shows the Gini importance derived from the Random Forest algorithm, which is a measure of how much each input variable contributes to the overall classification accuracy. It shows that the difference between the radar backscatter bands (quotient) is an extremely important variable in the classification. MNDWI and S-2 red-edge and shortwave infrared bands were also important. Red-edge dependent NDVI was more important than NDVI, and the sum average GLCM bands of both S-1 VV and VH polarizations were also important. The top-ranked variables in the classification were the quotient (VH-VV), B5, B2, B11, B12, MNDWI (Green-SWIR1)/(Green + SWIR1), B4, ratio (VH/VV), B3, B6, B7, VH, NDVI, VV, and EVI. Here, we elaborate on the mechanisms and patterns of influence associated with these variables. The quotient (VH-VV) and the ratio VH/VV are derived from the Sentinel-1 backscatter values, and they helped enhance the separability between classes with different vegetation densities or moisture content. These variables were particularly useful in distinguishing between vegetated areas, such as forests, cocoa, and plantations, and non-vegetated areas, like Bare land and Settlements. Spectral bands B2 (blue), B3 (green), B4 (red), and B5 (near-infrared) from Sentinel-2 were essential in differentiating between land cover types such as vegetation, water, and built-up areas. For instance, the blue band (B2) was effective in identifying water bodies, while the green (B3) and red (B4) bands helped differentiate between vegetation and built-up areas. The mid-infrared bands B11 and B12 were valuable for detecting variations in soil and vegetation moisture content. These bands proved beneficial in separating classes like Bare land, Shrubland, and Cropland from other vegetation classes with higher moisture content. MNDWI [(Green-SWIR1)/(Green + SWIR1)] is a spectral index that enhances the contrast between water and other land cover types. This index was particularly useful in accurately delineating Water class areas and minimizing confusion with other land cover types, such as Bare land and Settlements. The Normalized Difference Vegetation Index (NDVI) and Enhanced Vegetation Index (EVI) are widely used spectral indices for monitoring vegetation health and vigor. In our classification, these indices significantly contributed to the accurate identification of vegetation-related classes such as Forest, Cocoa, Plantation, Shrubland, and Cropland. They provided insights into the density and greenness of vegetation, which helped differentiate between various vegetation types. By incorporating these sensing variables and their corresponding mechanisms or patterns of influence, our LULC classification process was able to achieve high accuracy in delineating and differentiating between the various land use and land cover classes in the study area.

**Figure 6:**
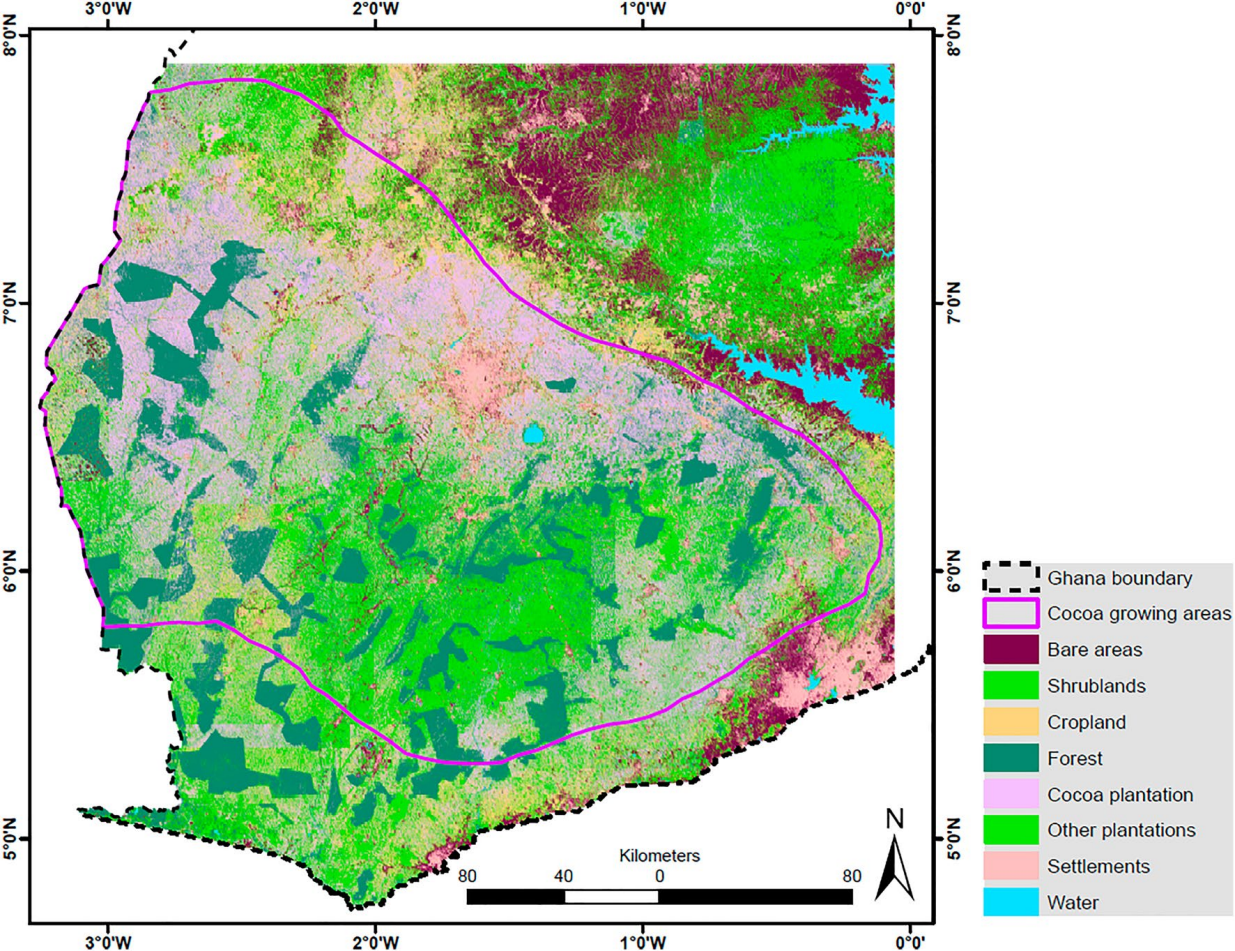
Land use and land cover map for the cocoa growing area in southern Ghana.

**Figure 7:**
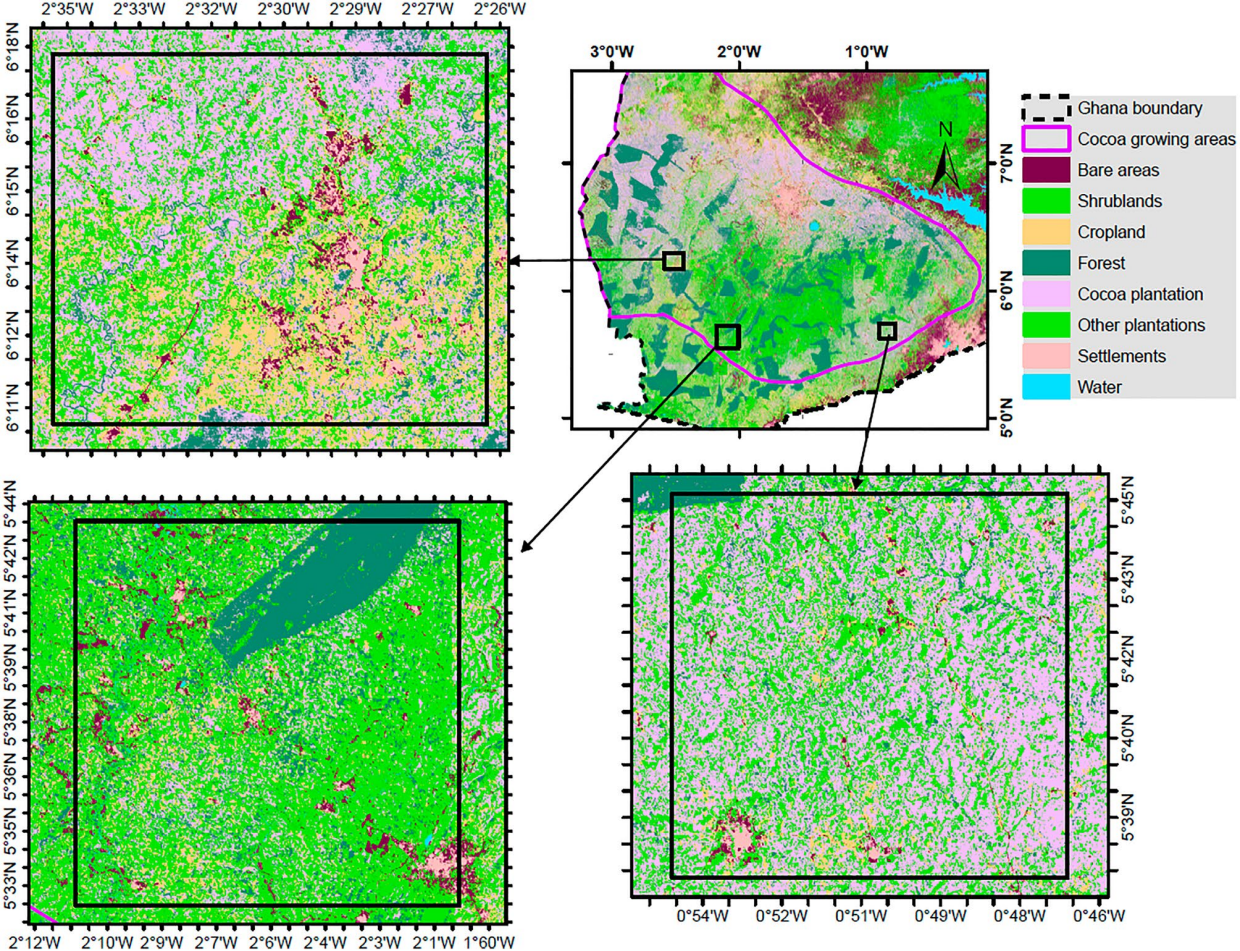
Zoomed-in maps of the cocoa growing area LULC map in southern Ghana.

Figure 6 shows the LULC map, while Table 4 provides the area coverage of the classes. The map shows that the forest, settlement, and water classes were well delineated due to the clear boundaries they exhibit. The cocoa class is dominant, with an area coverage of about 37%. The shrub and cropland classes are mostly in the north and south, respectively. The other plantations are concentrated in the middle and southwestern portions of the study area. These patterns are expected as many staples are grown in the northern part of the study area while the southern part has more plantations. Figure 7 shows zoomed-in portions of the map, from which the patterns are made clear.

**Table 4:** Area coverage of the land use/ land cover classes.

LULC class	Bare	Shrubland	Cropland	Forest	Cocoa	Plantation	Settlement	Water
Area (km^2^)	4316	6443	7331	8085	22,126	9771	879	125
% Area	7.31	10.91	12.41	13.69	37.45	16.54	1.49	0.21

### Surface water availability

**Figure 8:**
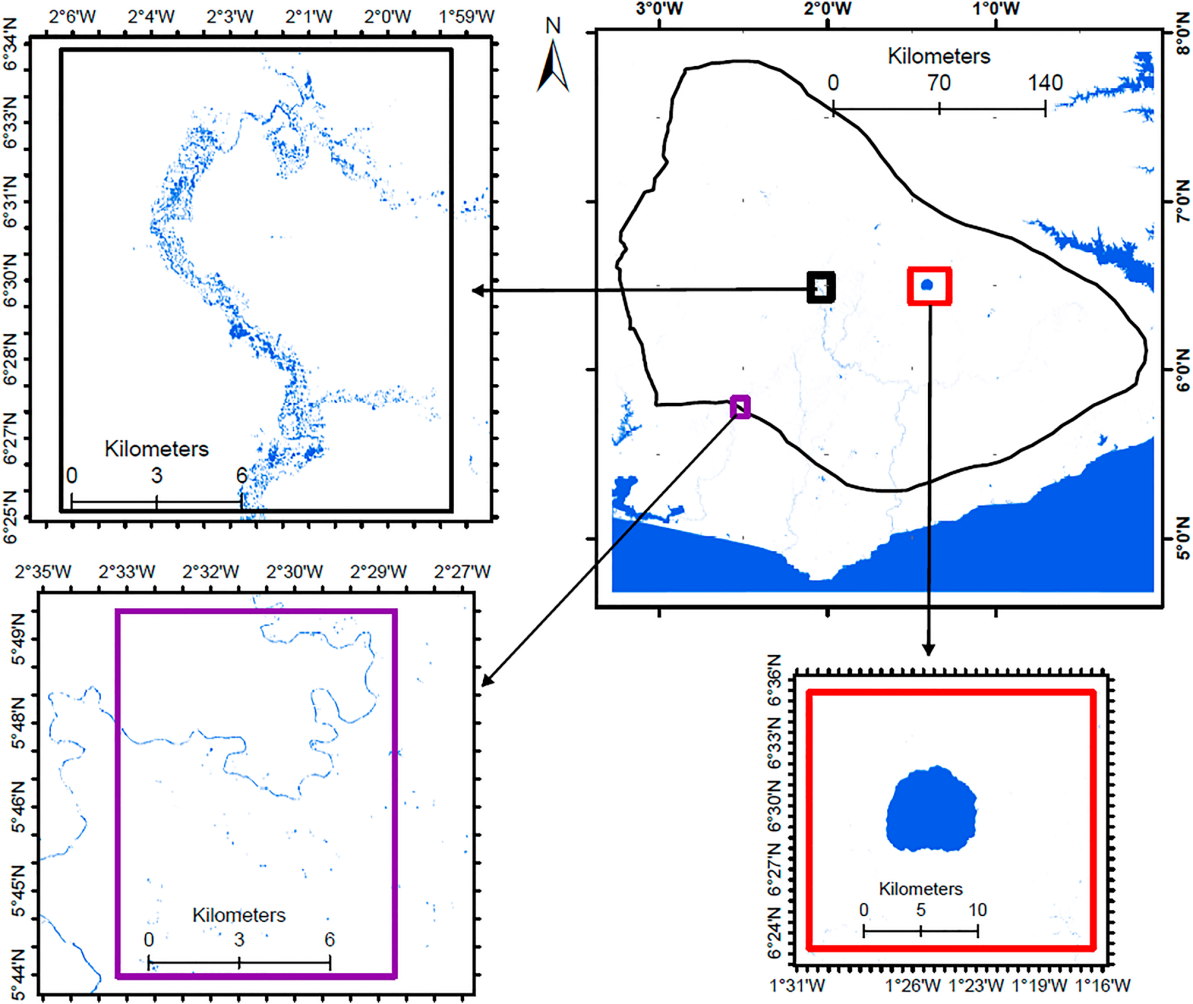
Water bodies in the cocoa-growing areas of Southern Ghana.

Figure 8 shows the water bodies within the cocoa-growing areas. The analysis revealed 125 km^2^ of surface water resources (rivers, lakes, dams, etc.). This includes Lake Bosomtwe, which has an area of 49 km^2^, and the Barekese Dam, which supplies water to most of Kumasi. It includes water bodies and ponds along rivers created due to illegal mining, locally known as galamsey, small reservoirs, lakes, and lagoons. As the water body class was accurately delineated, it is believed that this is a good reflection of the area of water bodies. It should, however, be noted that this could differ depending on seasonality.

### Groundwater assessment

#### Groundwater availability

**Figure 9:**
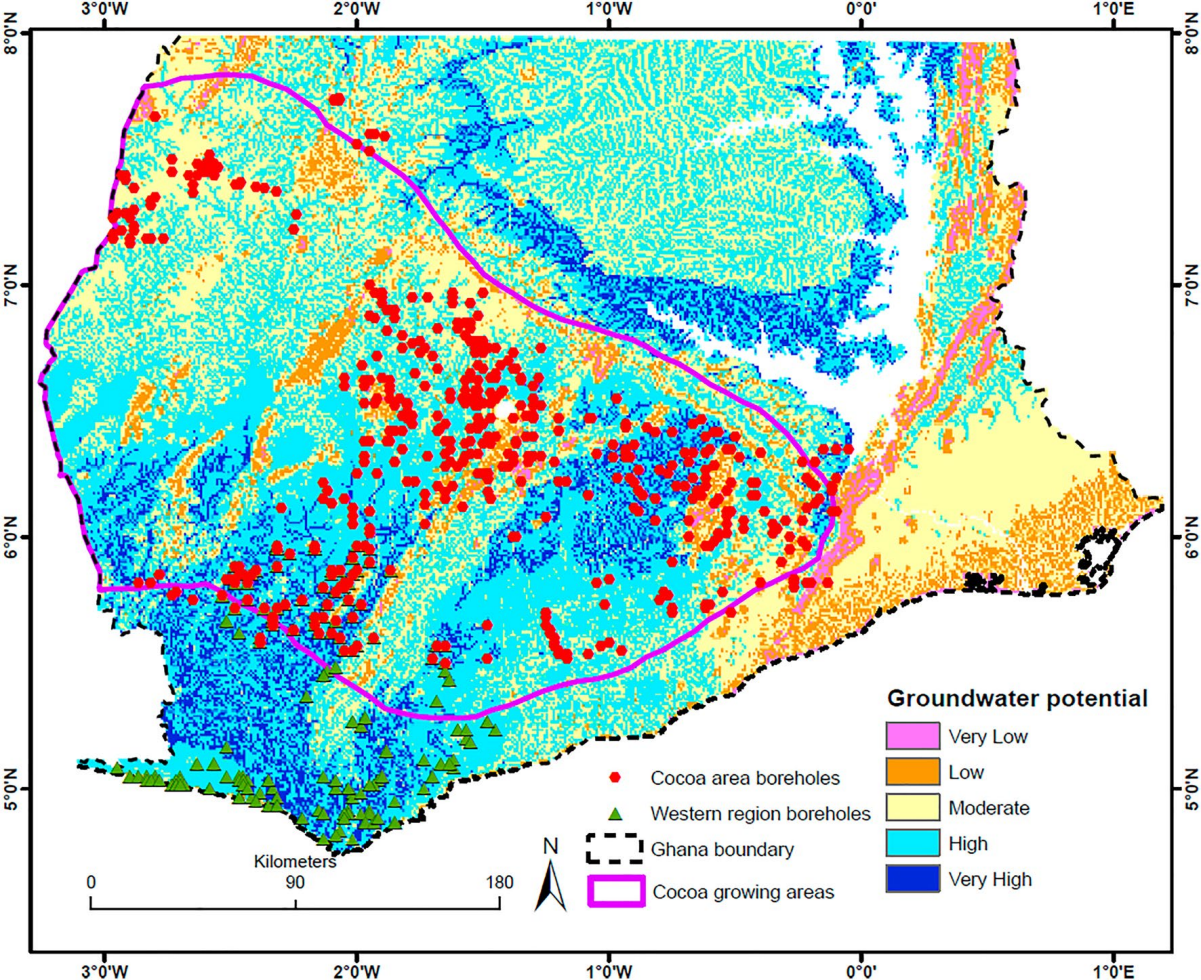
Results of MCA analysis combining all factors except borehole presence layer.

Figure 9 shows the result of the MCA overlaid with the locations of existing boreholes. A significant portion of the study area (~ 80%) has moderate to very high groundwater availability potential (Table 5). The areas where existing boreholes are located are predominantly classified as having high potential for groundwater availability, although some fall in the very low class. The reasonable correlation between existing borehole locations and high groundwater potential areas can be considered a measure of the reliability of the modeled groundwater availability potential map (qualitative validation). Further, the western region boreholes not included in the analysis fall in areas with high or very high groundwater availability potential. This means there is a high potential for obtaining groundwater in areas modeled as having high or very high potential.

**Table 5:** Percentage area under different groundwater availability potential for the two main MCAs.

GW availability potential classes	MCA results (% Area)
Very low	5.3
Low	16.5
Moderate	29.7
High	31.7
Very high	16.8

As earlier indicated, the downside of the MCA approach is that obtainable groundwater volumes cannot be determined from such an output. However, records of existing boreholes in each modeled potential zone can be used to infer the potential yields obtainable in each zone.

Table 6 summarises the characteristics of boreholes found in each potential zone. It presents the number, minimum, maximum, mean, and standard deviation of existing boreholes in each zone. The table shows a high (obtainable) mean yield of 90.7 l/min for the very high zone, although it also records the highest standard deviation (157 l/min), indicating high variability in obtainable yields. A significant proportion (> 50%) of boreholes fall within moderate and high potential zones and have relatively higher standard deviations than very low and low potential zones. Low groundwater potential zones recorded the lowest (obtainable) mean yield of 58 l/min, with very low, moderate, and high potential zones having mean yields of about 65 l/min. Considering that a greater part of the study area is categorized as moderate or high potential zones, the mean yield in these zones (i.e. 65 l/min) can be used as a good basis to estimate the potential groundwater available or obtainable in the study area.

**Table 6:** Summary statistics of existing boreholes in the modelled groundwater availability zones (MCA).

Potential class	Number of boreholes	Min. yield (l/min)	Max yield (l/min)	Mean yield (l/min)	Standard deviation
Very low	17	12.6	120	66.3	33.2
Low	69	10	138	58.2	33.6
Moderate	135	5	750	65.5	92.3
High	150	3	650	64.0	70.7
Very high	55	11.3	1200	90.7	157.6

#### Groundwater accessibility

Groundwater accessibility could not be explored due to limited information in the borehole dataset. In previous studies, e.g., Forkuor et al.^14^, information on static water level (SWL) was used to infer groundwater accessibility. In this case, although a few of the boreholes (< 10%) had information on SWL, they also had duplicate records with the same coordinates but different SWL values. In the absence of SWL data, future studies could explore possibilities of inferring accessibility from the main geological formations, e.g., typical depths to which groundwater is accessed.

### Evaluation of groundwater availability based on anomaly analysis: insights from GRACE and GLDAS data

**Figure 10:**
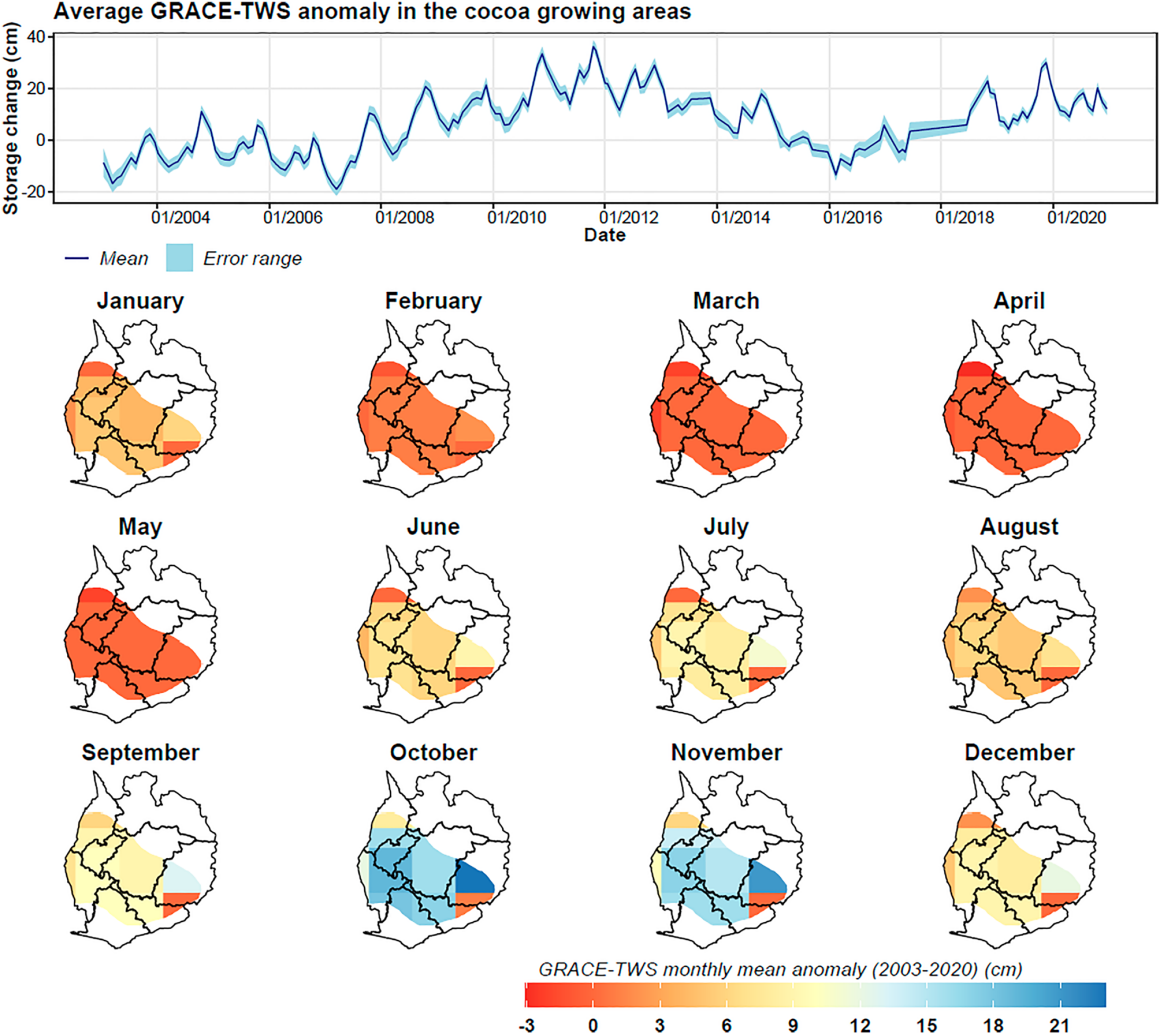
GRACE terrestrial water storage anomalies in the cocoa growing areas, showing temporal variability of the zonal mean (dark blue) and uncertainty bands (light blue), and the spatial monthly mean maps for the period 2003–2020.

**Figure 11:**
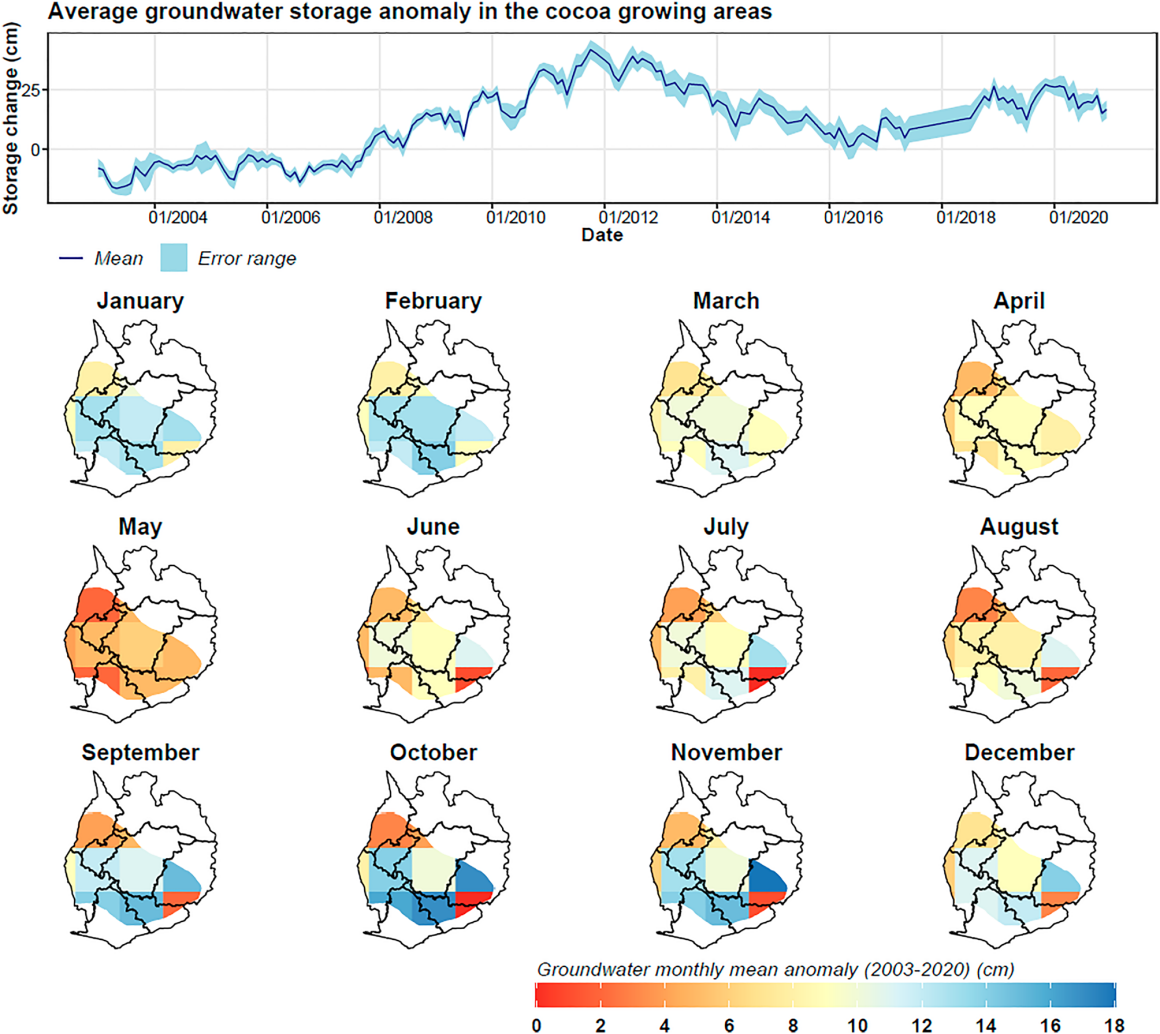
Derived groundwater storage anomalies based on the mass balance equation in the cocoa growing areas, showing temporal variability of the zonal mean (dark blue) and uncertainty bands (light blue), and the spatial monthly mean maps for the period 2003–2020.

**Figure 12:**
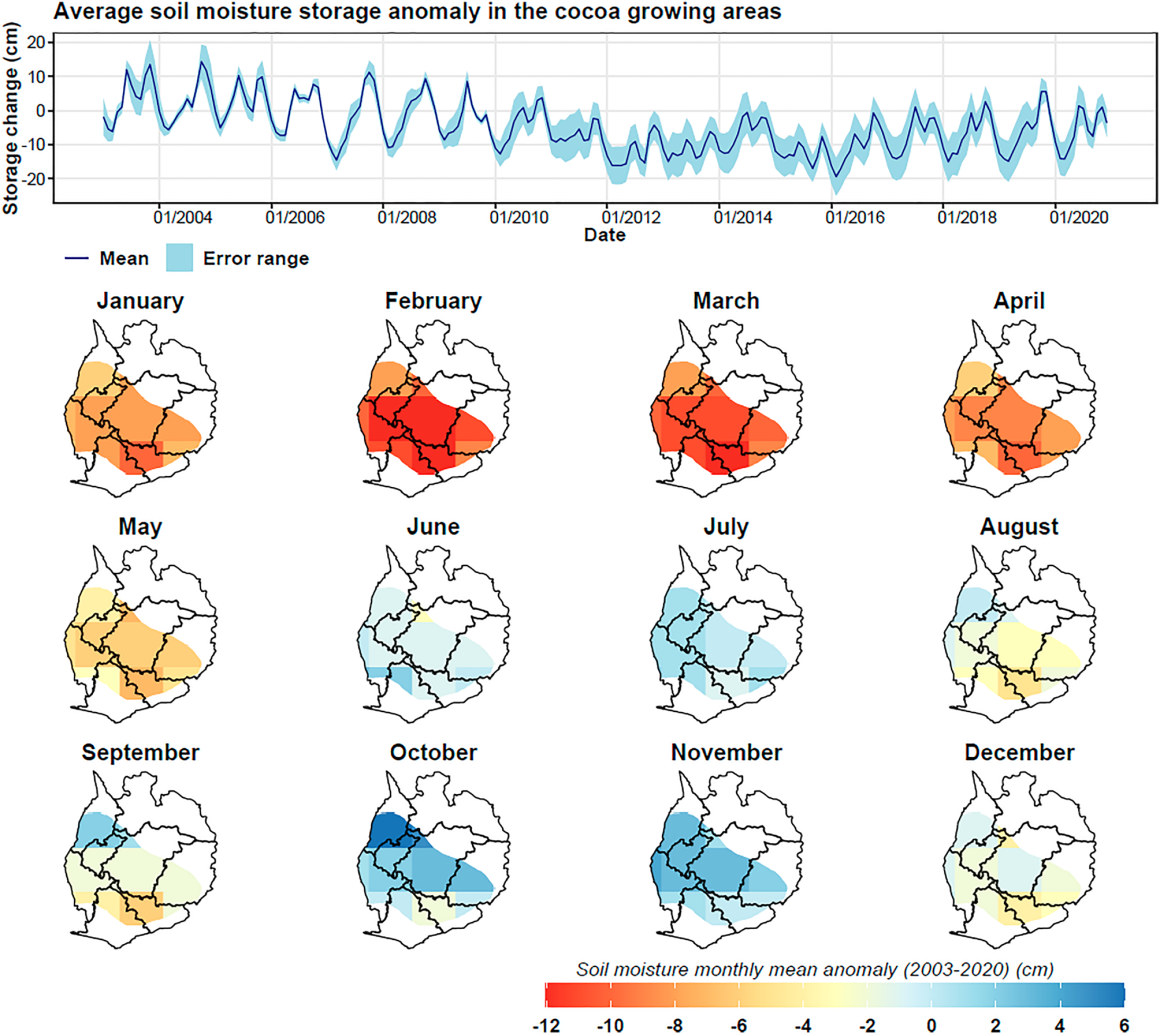
Soil moisture storage anomalies based on the mean of land surface models (Noah, VIC, and CLSM) in the cocoa growing areas, showing temporal variability of the zonal mean (dark blue) and uncertainty bands (light blue), and the spatial monthly mean maps for the period 2003–2020.

**Figure 13:**
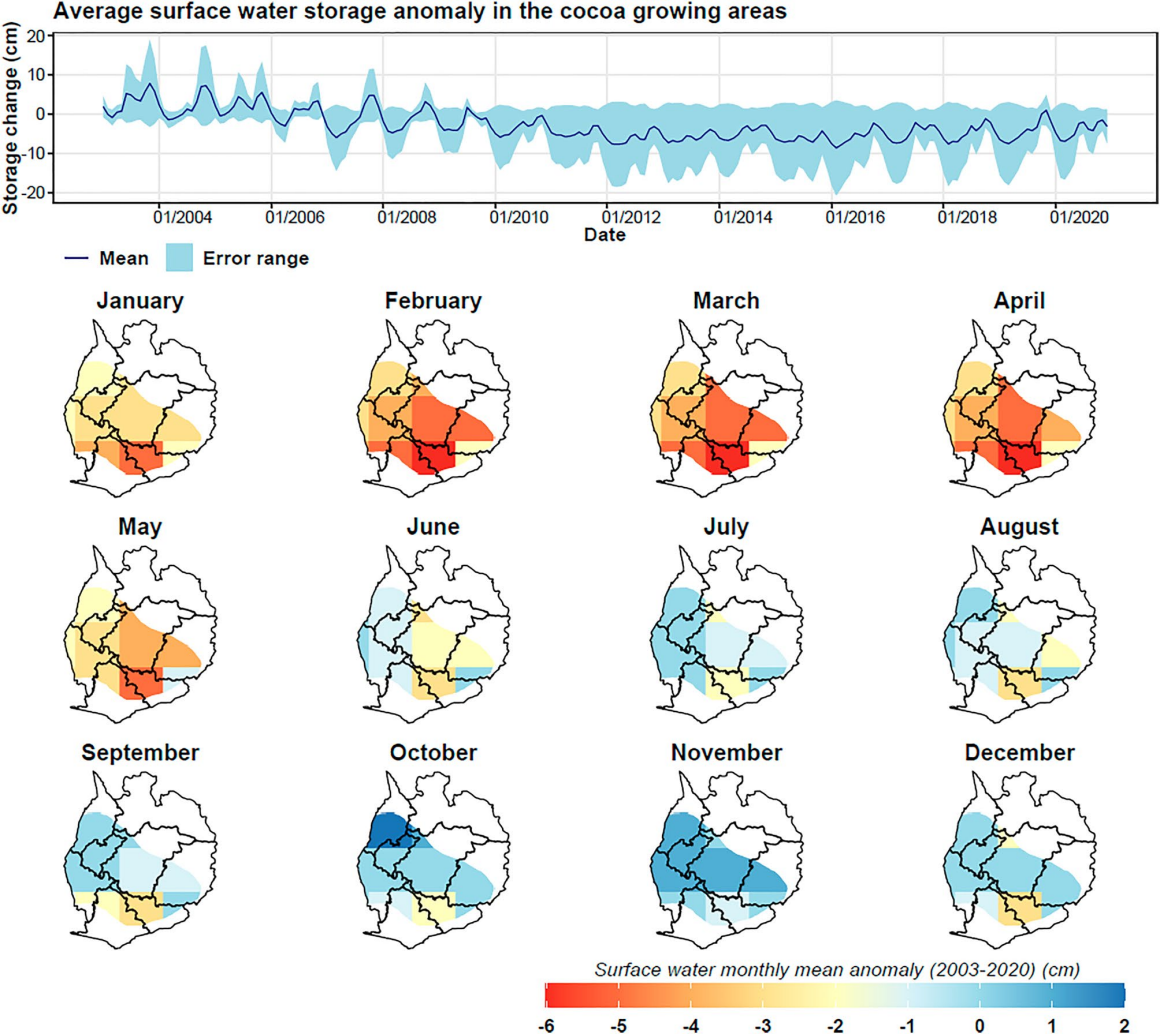
Surface water storage anomalies based on the mean of land surface models (Noah, VIC, and CLSM) in the cocoa growing areas, showing temporal variability of the zonal mean (dark blue) and uncertainty bands (light blue), and the spatial monthly mean maps for the period 2003–2020.

The hydrographs generated for each water storage component—both from GRACE and GLDAS datasets—indicate distinct temporal and spatial variability in water storage (Figs. 10, 11, 12, and 13). The time-series analysis of zonal mean GRACE terrestrial water storage anomalies from 2003 to 2020 reveals a modest overall increase in water storage at a rate of 0.003 cm/month. Yet, a nuanced inspection of these hydrographs uncovers more intricate fluctuations (Fig. 10). Notably, water storage anomalies increased by 0.0104 cm/month and 0.015 cm/month during 2003–2012 and 2016–2020, respectively. Conversely, there was a decline of − 0.018 cm/month between 2013 and 2015.

The groundwater storage anomalies exhibit similar patterns (Fig. 11), correlating well with terrestrial water storage anomalies (R^2^ = 0.75). The overall groundwater storage anomaly increased by 0.005 cm/month from 2003 to 2020. Interestingly, this finding has implications for cocoa irrigation in Ghana. The increase in groundwater storage suggests that groundwater could be a reliable source for irrigation, particularly during periods when surface water is scarce.

While the temporal data from GRACE indicates overall increases in water storage, soil moisture and surface water show a different trajectory. Contrary to GRACE terrestrial water storage (TWS) and groundwater anomalies, soil moisture and surface water experienced a downward trend, with rates of − 0.002 cm/month and − 0.0013 cm/month respectively over the 2003–2020 period (Figs. 12 and 13). This decrease, particularly since 2010, warrants concern for cocoa production as reduced soil moisture and surface water availability can lead to water stress in cocoa plants.

Spatial analysis further substantiates these trends. GRACE TWS anomalies were generally lower during the dry months (January-May) while rising significantly during the wet season (June-November). On the other hand, soil moisture and surface water anomalies were typically below normal from December to May, correlating with the dry season. This seasonal variability implies that irrigation may be most crucial during these deficit periods.

The observed decrease in soil moisture and surface water anomalies, especially during 2010–2020, has significant implications for cocoa production in Ghana. Water scarcity during the dry season, as evidenced by our data, necessitates the adoption of sustainable irrigation strategies. Given the increased groundwater storage anomalies, the implementation of groundwater-based irrigation systems appears to be a viable adaptive strategy for enhancing the resilience of cocoa farms against climate-induced water stress.

### Limitations and future research

The study's limitations mainly pertain to the completeness and quality of data used in the two main analyses. In summary, these are:

- *Ground truth/reference data* The ground truth data used for the classification and validation of the LULC map were obtained from Google Earth and other online platforms. Whereas for certain classes (e.g., water, settlements), this approach is suitable, the same cannot be said for other classes, such as cocoa plantations which are difficult to discern even on a high-resolution image. In this study, the locations (centroids) of cocoa farms were obtained from the website of cocoalife, which served as the basis for extracting samples. A field campaign is recommended in the future to confirm the locations of a sample of the data currently online. Other LULC classes, such as shrubland and cropland, would also benefit from such a campaign.
- *Groundwater occurrence influencing factors* The groundwater availability assessment could have benefitted from additional data/factors influencing groundwater occurrence. For example, spatial layers of regolith thickness and transmissivity obtained from existing boreholes can improve the accuracy of the modeling. However, such attributes were not found in the borehole data available to authors. This constitutes a limitation in the obtained results, which should be utilized with these limitations in mind.
- *Borehole data* The borehole data that was available had two critical limitations. The first relates to multiple records having the same geographic coordinates but different yields. This is believed to have contributed to the poor results obtained for the regression-based analysis and spatial interpolation approach (see Appendix A3). The feasible solution to this problem will be to conduct ground visits and use the drilling date to re-establish the coordinates of the boreholes. The second limitation relates to the lack of information on SWL. Although there is information on “total depth”, it applies to just about 30% of the records. This limited the possibility of conducting an accessibility analysis.
- It is important to acknowledge the limitations of our groundwater potential assessment. Firstly, our analysis did not directly incorporate aquifer characteristics, such as depth and type (e.g., confined or unconfined aquifers), due to the limited availability of such data. These factors play a significant role in determining the hydraulic head observed in boreholes and characterizing groundwater systems. Secondly, our validation approach relied on the correlation between existing borehole locations and high groundwater potential areas, which serves as a preliminary validation and should be interpreted with caution. Future studies should seek to refine the groundwater potential assessment by incorporating more detailed data on aquifer characteristics, as well as employing more robust validation methodologies. Despite these limitations, our study provides a valuable starting point for understanding groundwater potential in the cocoa-growing regions of Ghana and offers decision-support maps that can inform planning and investment in climate-smart cocoa irrigation.
- *Weighting in MCA* Ideally, influencing factors in an MCA are combined to reflect their relative importance. This implies the assignment of variable weights to the factors under consideration. In this study, we used equal weighting as a preliminary approach due to the lack of sufficient local knowledge or data to guide the assignment of specific weights to each factor. This equal weighting scheme may not accurately reflect the true influence of each factor on groundwater availability potential, and the assignment of variable weights can lead to different results. For future research, we propose employing a more sophisticated weighting scheme that accounts for the relative importance of each factor, as informed by the integration of local geological data and expert opinions. One possible approach to achieve this is through the Analytical Hierarchy Process (AHP), which facilitates the elicitation of expert knowledge to derive a set of weights for each factor based on their relative importance. By incorporating local geological information and applying a more refined weighting scheme, future research can provide a more accurate assessment of groundwater availability potential, contributing to more informed decision-making regarding the implementation of climate-smart cocoa irrigation practices in Ghana.
- While our study provides valuable insights into the potential of surface and groundwater resources for climate-smart cocoa irrigation in Ghana, we acknowledge several limitations. One major constraint is the lack of detailed investigation into the dynamic characteristics of surface and groundwater resources in the study area. Understanding the temporal and spatial variations in water availability, recharge rates, and interactions between surface and groundwater is crucial for developing effective irrigation strategies. Although our current assessment focuses on the availability and potential of surface and groundwater resources, it does not account for the complex hydrological processes governing water resource dynamics in the region. As a result, our study may not fully capture the implications of these processes on the feasibility of implementing sustainable cocoa irrigation practices. To address this limitation, we propose several avenues for future research. First, a comprehensive hydrological modeling study, incorporating both surface and subsurface water dynamics, could provide a more accurate representation of the water resource potential in the cocoa-growing regions. Second, long-term monitoring of surface and groundwater resources, including water quality and quantity assessments, would contribute to a better understanding of the temporal and spatial dynamics of these resources. By conducting such investigations, future research can build on our findings and provide a more comprehensive understanding of water resources in the region, ultimately contributing to the development of effective and sustainable cocoa irrigation strategies.

## Conclusion

This study conducted a preliminary analysis to determine surface and groundwater availability for cocoa irrigation in the cocoa-growing areas of Ghana. Two analyses were conducted—land use/land cover from 10 m resolution S-1 and S-2 and groundwater availability assessment by combining seven groundwater occurrence influencing factors in an MCA. Eight LULC classes were considered: bare areas, cropland, shrubland, forest, cocoa plantation, other plantations, settlements, and water. Rainfall, recharge, elevation, slope, TWI, geology, and temperature are combined to assess groundwater availability. The reliability of the MCA result was assessed with the location of boreholes, while reference data from Google Earth were used to validate the LULC map.

The LULC analysis yielded an overall accuracy of 96.8%. Good classification accuracies were obtained for all classes, although some confusion was found among the vegetative classes. For example, there was confusion between cocoa on the one hand and cropland and shrubland on the other. Forest areas were also misclassified as cocoa or other plantations. Image bands and derivatives that were found to be important and contributed to the high accuracy obtained include the shortwave infrared and red-edge bands, MNDWI, red-edge dependent NDVI, and the difference between radar backscatter bands (quotient). Within the cocoa growing area, the analysis revealed a total area of 22,126 km^2^ and 125.2 km^2^ for cocoa plantations and water bodies, representing the highest (37.5%) and lowest (0.2%) coverages, respectively.

The MCA analysis showed that about 80% of the study area has moderate to very high groundwater availability potential. The overlay between the MCA output and existing borehole location revealed a reasonable correlation between the two, with about 80% of existing boreholes falling in moderate to very high potential areas. Boreholes that fell in very high potential areas had the highest mean yield of 90.7 l/min, while the lowest mean of 58.2 l/min represented boreholes in low groundwater availability potential areas.

These results notwithstanding, the data and methods employed in this study have a few limitations that must be addressed in follow-up studies. Some minimal fieldwork is required to verify the locations of some LULC classes (e.g., cocoa) to improve the quality of reference data used for the LULC classification. Fieldwork is also required to re-establish the coordinates of borehole locations and resolve the problem with duplicate records. On the other hand, future studies can test the possibility of downscaling GRACE data to a reasonable spatial resolution (e.g., 5 km) that can form the basis of groundwater availability assessment in cocoa-growing areas.

### Supplementary Information

Supplementary Information.

## Data Availability

The datasets that support the findings of this study are available from the corresponding author upon reasonable request.
