# Supplementary material for: Integrating GIS and remote sensing for land use/land cover mapping and groundwater potential assessment for climate-smart cocoa irrigation in Ghana
Source: Sci Rep. 2023 Sep 25;13:16025. doi: 10.1038/s41598-023-43286-5 (PMC10520077; doi:10.1038/s41598-023-43286-5)
Supplement: Supplementary file 1 — Supplementary Information. [file 41598_2023_43286_MOESM1_ESM.docx]

# **Appendices**

**Appendix A1.** List of bands and derivatives used for LULC classification

**Table A1.1.** List of bands and derivatives used for LULC classification

| **Sensor** |  | **Bands, indices, or parameters** | **Definition** |
| --- | --- | --- | --- |
| Sentinel-1 | Polarization | VV | Vertical transmit-vertical channel |
|  |  | VH | Vertical transmit-horizontal channel |
|  | Indices | VH-VV (Laurin et al., 2018) | Quotient |
|  |  | VH+VV (Laurin et al., 2018) | Product |
|  |  | VH/VV (own construct) | ratio |
|  | Textural (GLCM) | 18 parameters each computed from the two backscatter bands (VV, VH) | https://developers.google.com/earth-engine/apidocs/ee-image-glcmtexture |
| Sentinel-2 | Multispectral Bands | Band 2 | Blue, 490 nm |
|  |  | Band 3 | Green, 560 nm |
|  |  | Band 4 | Red, 665 nm |
|  |  | Band 5 | Red edge (Rede 1), 705 nm |
|  |  | Band 6 | Red edge (Rede 2), 749 nm |
|  |  | Band 7 | Red edge (Rede 3), 783 nm |
|  |  | Band 8 | Near Infrared (NIR), 842 nm |
|  |  | Band 8A | Near Infrared (NIR), 865 nm |
|  |  | Band 11 | SWIR1, 1610 nm |
|  |  | Band 12 | SWIR2, 2190 nm |
|  | Vegetation Indices | NDVI (Rouse et al., 1973) | (NIR–Red)/(NIR+Red) |
|  |  | NDVI_RE5_ (Own construct) | (Rede1–NIR)/(Rede1+NIR) |
|  |  | NDVI_RE6_(Own construct) | (Rede2–NIR)/(Rede2+NIR)) |
|  |  | EVI | (2.5x(NIR-Red))/(NIR+6Red-7.5Blue+1) |
|  |  | EVI12 | (2.5x(NIR-Red))/(NIR+2.4Red+1) |
|  |  | SAVI | ((1+L)(NIR-Red))/(NIR+Red+L); L=0.5 |
|  |  | NDBI | (SWIR1-NIR)/(SWIR1+NIR) |
|  |  | MNDWI | (Green-SWIR1)/(Green+SWIR1) |
|  |  | LSWI | (NIR-SWIR1)/(NIR+SWIR1) |

**Table A1.2.** Snapshots from Google Earth depicting the eight LULC classes

| **Cropland** | 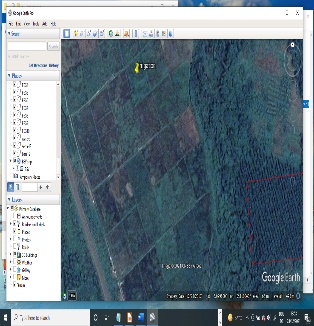 | 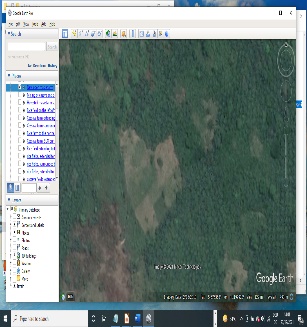 |
| --- | --- | --- |
| **Shrubland (closed to open)** | 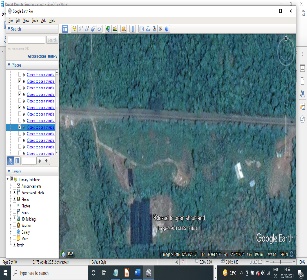 | 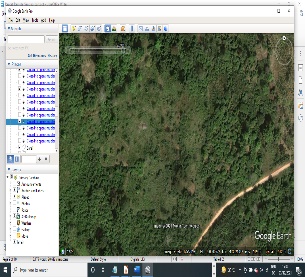 |
| **Forest (evergreen)** | 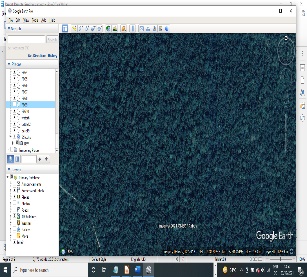 | 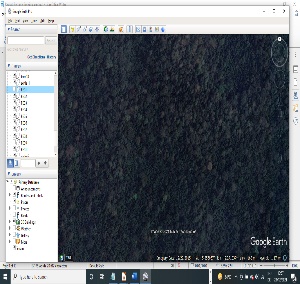 |
| **Cocoa** | 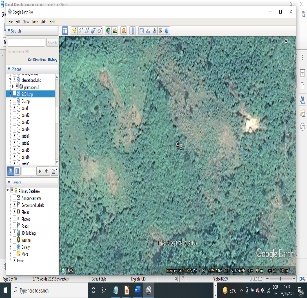 | 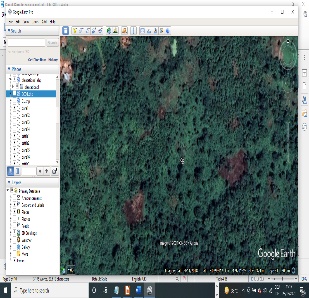 |
| **Plantations (rubber, palm, orange, teak)** | 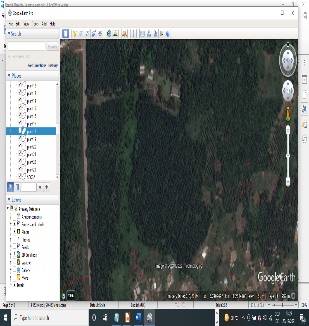 | 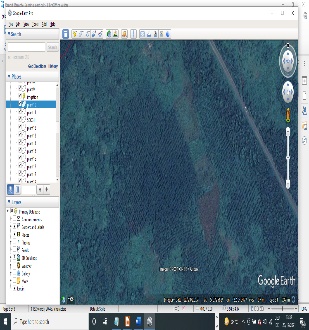 |
| **Settlement** | 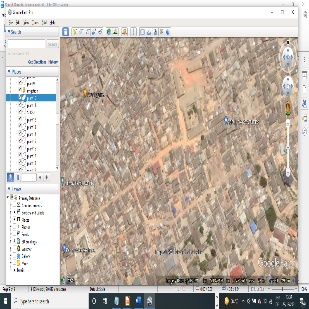 | 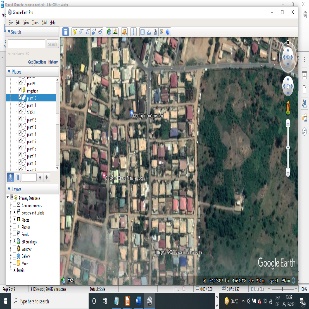 |
| **Water** | 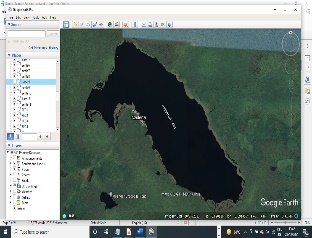 | 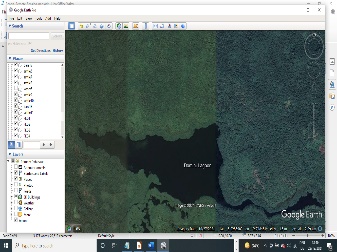 |

**Appendix A2. Spatial Interpolation**


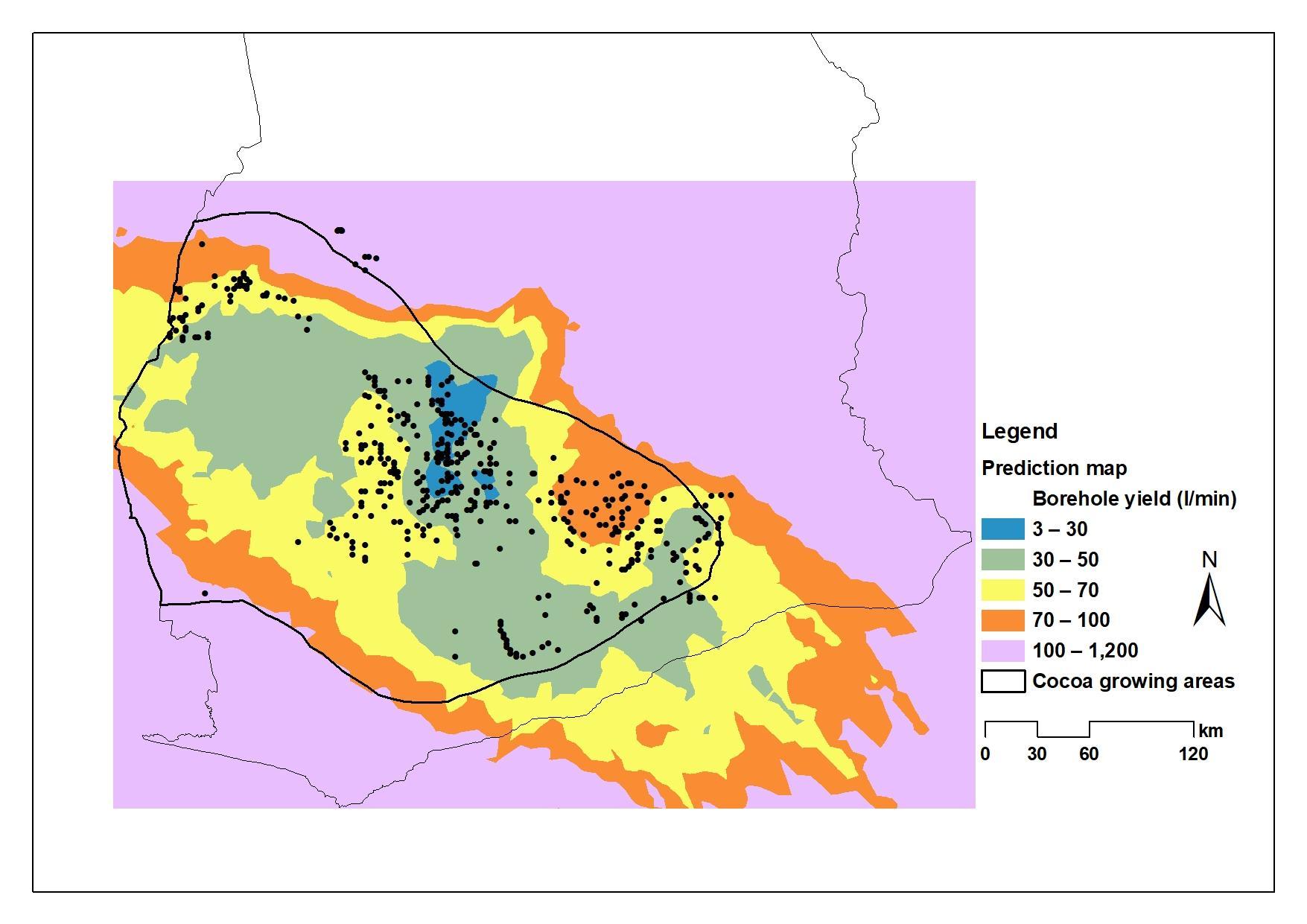


**Figure A2.1.** Spatial distribution of borehole yield based on ordinary Kriging.

Spatial interpolation is a technique that permits values of sampled locations to be used to predict corresponding values for unsampled locations. In this case, existing borehole locations with yield information can be used to determine potential yields where there are no boreholes. Although groundwater availability cannot be solely inferred from borehole yield, it is the only attribute in the borehole dataset that can be used as a proxy for groundwater availability.

The spatial interpolation method employed in this study was Kriging (ordinary). Kriging estimates values at unsampled locations based on a surface model generated from values at sampled locations. It was chosen because it has several advantages over other spatial interpolation methods, such as spline and inverse distance weighting [40,41].

The borehole locations dataset was first explored to check for normality and trends ([42]. A log transformation was applied to the data to ensure normality, and a second-order polynomial was applied for trend removal. The transformed data were used to estimate a semi-variogram model based on which borehole yield at unsampled locations was predicted.

Table A2.1 shows the estimated (semi-variogram) model (left) used to predict borehole yield at unsampled locations. The table also shows the regression equation, and the prediction accuracy estimates obtained. For a model that delivers accurate prediction, the mean prediction error is expected to be close to “0”, with the root-mean-square standardized prediction error close to 1 [43]. Based on these assumptions and judging from the prediction estimates obtained (Table A2.1), the developed model has low predictive power. The root mean square error and average standard error are also deemed very high, considering a mean borehole yield of 60 l/min (data with duplicate records) or 67 litres/min (records with duplicates averaged). The prediction and QQplot shown in Figure A2.2 also confirm the model's poor predictive power. The prediction plot shows a poor relationship between measured and predicted values.

As discussed in the previous section, the poor predictive power of the model can be attributed to the underlying borehole data that contained duplicate records. Averaging the yield information for multiple records with the same geographical information may have introduced errors because of the high variability in the yield values for some duplicate records (e.g. 7, 15, 20, 40, 50, 60, 80, 150).

**Table A2.1. Left:** Semi-variogram model based on which yield was predicted; right: prediction accuracy statistics from the model

| 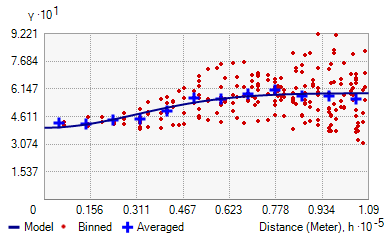 | **Regression function** | **‎0.203372 * x + 46.01** |
| --- | --- | --- |
|  | 0 | 0 |
|  | 0 | -0.2 |
|  | 0 | 68.76 |
|  | 0 | 0 |
|  | 0 | 0.92 |
|  | 0 | 65.67 |


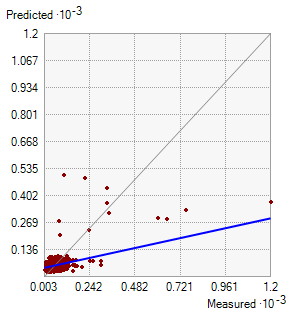

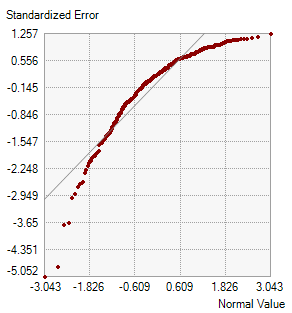


**Figure A2.2.** Prediction and QQ plot from the ordinary Kriging to spatially interpolate borehole yield data.

Despite the poor predictive power of the model, a borehole yield layer was predicted and shown in Figure A2.1. It shows that predictions remained in the range of borehole yields within the cocoa growing area in the original data. However, a significant portion of the area falls in the range of 3 to 50 l/min, which is lower than the mean of the dataset (either with duplicate records or with averaged values). Due to the poor prediction errors, this map may not be relied on for decision-making regarding groundwater potential for cocoa irrigation.

**Appendix A3. Regression-based approach**

The national-level borehole dataset was used as the primary data to determine groundwater availability. Yield is an important borehole attribute from which groundwater availability can be inferred. There is a high probability that high-yielding boreholes will be situated in areas of high groundwater availability. Therefore, this approach adopted a regression-based method to explain the variability in borehole yield using environmental predictors. In other words, the method aimed to develop a regression equation between borehole yield and variables that influence it and subsequently use the Equation to predict borehole yield over the entire study area. Regression-based approaches are well-grounded in literature and have been used severally to predict, for example, groundwater recharge [44], groundwater level [45], crop yield [46] soil properties [21] and above-ground biomass [47].

In this study, the predictors considered were: (i) groundwater recharge, (ii) geology, (iii) elevation, (iv) slope, (v) topographic wetness index, (vi) NDVI, (vii) precipitation and (viii) temperature. Section 3.1 details how these predictors were obtained. Borehole yield is considered the dependent variable (response), and the predictors as independent variables.

The regression analysis was performed using the Random Forest (RF) Machine Learning Algorithm (MLA) [25]. RF belongs to the family of ensemble MLAs that predict a response from a set of predictors by growing a large number of decision trees (forest) and averaging the values predicted by all trees as the final result. Each tree in the forest is independently constructed using a unique bootstrap sample of the training data. RF is preferred over standard tree-based models because it is less sensitive to noise in the training data and produces more accurate predictive models. Comparative studies involving other MLAs (e.g. SVR, ANN, etc.) also showed RF's superior performance [48,49]. RF is robust against data redundancy and nonlinearity and can handle various predictors with different properties and values [48]. An important feature of RF is that it enables a determination of the relative importance of different predictors, which is essential for understanding the data and deciding on data investment.

Before running the model, the borehole locations were used to extract the corresponding data from all predictors. The resulting data were randomly split into two parts – 75% for model training and 25% for model validation. The RF model was run within the caret package in R [50]. Caret allows key RF modelling parameters such as the number of trees to be grown in the forest (ntree) and the number of randomly selected predictors (mtry) to be tuned and the best parameters selected. Parameter tuning was set using the grid search method and tenfold cross-validation with five repetitions. This enables an assessment of multiple combinations of the parameters against accuracy measures (e.g., RMSE, MAE, R^2^).

The model tuning process results revealed an R^2^ and RMSE of 0.3 and 77.4, respectively, with two optimal predictors randomly selected at each split. Based on this, the final model was found to explain only 22% of the variance in the yield data (see Table A3.1). Validation of the model using the 25% test data yielded an extremely low R^2^ of 0.05, although the RMSE and MAE were better than those obtained in the cross-validation (using the training data). This suggests some inconsistency in the underlying data and raises concerns about the model's predictive power. Although adding more predictors can improve the accuracy measures, the duplicate record in the original data is a possible cause of the low accuracy measures.

**Table A3.1.** Random Forest Model Specifications

| ***Random Forest – Cross-validation***  *322 samples; 8 predictor*  ***Resampling: Cross-Validated (10 fold, repeated five times)***  *mtry RMSE Rsquared MAE*  *2 77.36470 0.3330178 41.03266*  *5 79.78308 0.3319585 41.93743*  *8 80.51186 0.3332254 42.06361*  *RMSE was used to select the optimal model using the smallest value.*  *The final value used for the model was mtry = 2.*  ***Final RF model***  *Call:*  *randomForest(x = x, y = y, mtry = min(param$mtry, ncol(x)), importance = ..1)*  *Type of random forest: regression*  *Number of trees: 500*  *No. of variables tried at each split: 2*  *Mean of squared residuals: 7604.619*  *% Var explained: 22.1*  ***Model Validation Based on 104 Test Samples***  *RMSE Rsquared MAE*  *45.81753801 0.05382376 32.17665579* |
| --- |

As indicated above, assigning the average yield to multiple records could have introduced significant errors that might have propagated through the regression analysis. Due to the low accuracy statistic, the regression equation was not applied to predict a borehole yield layer for the entire area.

Nonetheless, the variable importance plot (Figure A3.2) showed that recharge, climatic factors, elevation and NDVI were strong predictors of borehole yield and could be explored subsequently when accurate borehole records are available.


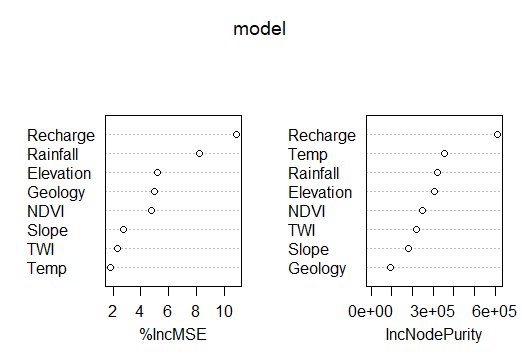


**Figure A3.2.** Variable importance plot from the random forest regression. Variables with high “InNodePurity” are high predictors of groundwater yield.
